# Manipulating Crystal Growth and Secondary Phase PbI_2_ to Enable Efficient and Stable Perovskite Solar Cells with Natural Additives

**DOI:** 10.1007/s40820-024-01400-w

**Published:** 2024-04-29

**Authors:** Yirong Wang, Yaohui Cheng, Chunchun Yin, Jinming Zhang, Jingxuan You, Jizheng Wang, Jinfeng Wang, Jun Zhang

**Affiliations:** 1 Institute of Chemistry, Chinese Academy of Sciences (CAS), Beijing, 100190 People’s Republic of China; 2 University of Chinese Academy of Sciences, Beijing, 100049 People’s Republic of China; 3 Nanjing University, Nanjing, 210023 People’s Republic of China; 4 Wuhan Textile University, Wuhan, 430200 People’s Republic of China

**Keywords:** Perovskite, Solar cells, Defect passivation, Biomass additives, Crystal orientation

## Abstract

In perovskite solar cells (PSCs), the inherent defects of perovskite film and the random distribution of excess lead iodide (PbI_2_) prevent the improvement of efficiency and stability. Herein, natural cellulose is used as the raw material to design a series of cellulose derivatives for perovskite crystallization engineering. The cationic cellulose derivative C-Im-CN with cyano-imidazolium (Im-CN) cation and chloride anion prominently promotes the crystallization process, grain growth, and directional orientation of perovskite. Meanwhile, excess PbI_2_ is transferred to the surface of perovskite grains or formed plate-like crystallites in local domains. These effects result in suppressing defect formation, decreasing grain boundaries, enhancing carrier extraction, inhibiting non-radiative recombination, and dramatically prolonging carrier lifetimes. Thus, the PSCs exhibit a high power conversion efficiency of 24.71%. Moreover, C-Im-CN has multiple interaction sites and polymer skeleton, so the unencapsulated PSCs maintain above 91.3% of their initial efficiencies after 3000 h of continuous operation in a conventional air atmosphere and have good stability under high humidity conditions. The utilization of biopolymers with excellent structure-designability to manage the perovskite opens a state-of-the-art avenue for manufacturing and improving PSCs.

**Supplementary Information:**

The online version contains supplementary material available at 10.1007/s40820-024-01400-w.

## Highlights

- The novel ionic cellulose derivative with cyano-imidazolium cation and chloride anion, designed from natural cellulose, promotes the crystallization process, grain growth, and orientation of perovskite in perovskite solar cells (PSCs).
- The use of ionic cellulose leads to the suppression of defect formation, reduction of grain boundaries, enhancement of carrier extraction, and inhibition of non-radiative recombination, resulting in a high power conversion efficiency of 24.71% in PSCs.
- The biopolymer-based approach enables excellent stability of PSCs, which maintain over 91.3% of their initial efficiencies after 3000 h in conventional air atmosphere.

**Supplementary Information:**

The online version contains supplementary material available at 10.1007/s40820-024-01400-w.

## Introduction

Perovskite materials used in solar cells have attracted ever-increasing interest, because of their low exciton binding energy, high carrier mobility, and excellent absorption coefficient [1–3]. Since the perovskite solar cells (PSCs) were prepared by Miyasaka in 2009, their power conversion efficiencies (PCEs) have been constantly broken [4–7]. The highest certified PCE value achieved to 26.1%, but it is still far below the theoretical limit of 33.7% in terms of Shockley–Queisser limit [8, 9]. Defects are prone to inertial formation during the preparation of perovskite, such as bulk defects (vacancies, interstitials, and antisites), interface defects, and grain boundary defects. The formation of defects not only results in an increase of the non-radiative recombination of the photogenerated carrier to dissipate energy, but also accelerates the ion migration, which obstructs the enrichment of electrons and holes at the electrodes and suppresses the increase of device open-circuit voltage (*V*_oc_) and fill factor (*FF*) [10–13]. In addition, excessive defects make the perovskite layer display a strong n-type or p-type character, which is diverse to the energy level alignment with the hole and electron transport layers [14]. Besides, excess PbI_2_ has been demonstrated to further improve efficiency by increasing carrier lifetime and suppressing halide vacancies, but it easily accumulates at grain boundaries to initiate the degradation of perovskite under light, moisture, and/or heat stresses [15–17]. Therefore, defect reduction and interface optimization are essential to improve the performance and stability of PSCs.

Additive engineering is one of the effective strategies to eliminate the defects and improve PCEs. Various additives have been tried in the past decade, including polymers [18], Lewis acids and bases [19], salts [20], ionic liquids [21, 22], etc. The additives, which form the strong interactions with perovskite, can effectively adjust the growth of perovskite crystals and regulate the defects. For example, a series of imidazolium-based ionic liquids have been used to improve the quality of large-area perovskite film via forming a new Pb-N bond between the ionic liquids and Pb^2+^ [23–25]. The PCEs increased to 23.5%. Different from small molecule passivators which have some disadvantages of high volatility, easy mobility, and limited functional groups, polymer additives have received intensive attention because of their numerous functional groups, long-chain effect, strong orientation capability, and excellent structural designability [26–28]. Yang et al*.* proposed an inter-grain cross-linking strategy to induce the orderly arrangement of perovskite precursor molecules along the polycarbonate chain [29]. The carbonate groups (–O–C(=O)–O–) on the main chain formed the strong interactions with CH_3_NH_3_^+^ and I^−^, thus both passivating the grain boundaries and regulating nucleation rate. Zhao et al*.* found that, owing to the hydrogen-bonding interactions, polyethylene glycol (PEG) slowed down the crystallization rate of perovskite, promoted uniform growth of grains, and improved the morphology of perovskite layer, and thus, the resultant PSCs exhibited the enhanced optoelectronic properties [30]. So far, after the utilization of the polymer additives, the PCEs of the PSCs were improved to 24%. However, the common petroleum-based polymers have limited functional groups. More attempts should be inspiringly taken to further improve the performance and stability of the PSCs, such as the utilization of the natural polymers.

Cellulose is the most available and abundant biopolymer in nature. It can be fractionated from all plants and crops, including wood, grass, cotton, bamboo, straws, bagasse, and so on [31–33]. Along cellulose chain, there are a large number of hydroxyl groups, which are regularly distributed. After a chemical modification, various groups have been covalently bonded on cellulose chains to precisely formulate the structures and properties [34–36]. Commercial cellulose derivatives, such as ethyl cellulose (EC) [37], hydroxyethyl cellulose (HEC) [38], hydroxypropyl cellulose (HPC) [39], and cellulose acetate (CA) [40], have been introduced into the fabrication process of PSCs. It is found that cellulose chains have good grain boundary localization ability, meanwhile passivate defects and promote grain growth via the hydrogen-bonding interactions between the hydroxyl groups and perovskite [27, 30]. However, the above cellulose derivatives (CDs) have only two types of functional groups, hydroxyl group, and ester group, which have a limited cooperation effect on perovskite. As a result, the reported cellulose additives exhibit an unremarkable impact on the PCEs enhancement of PSCs.

**Fig. 1:**
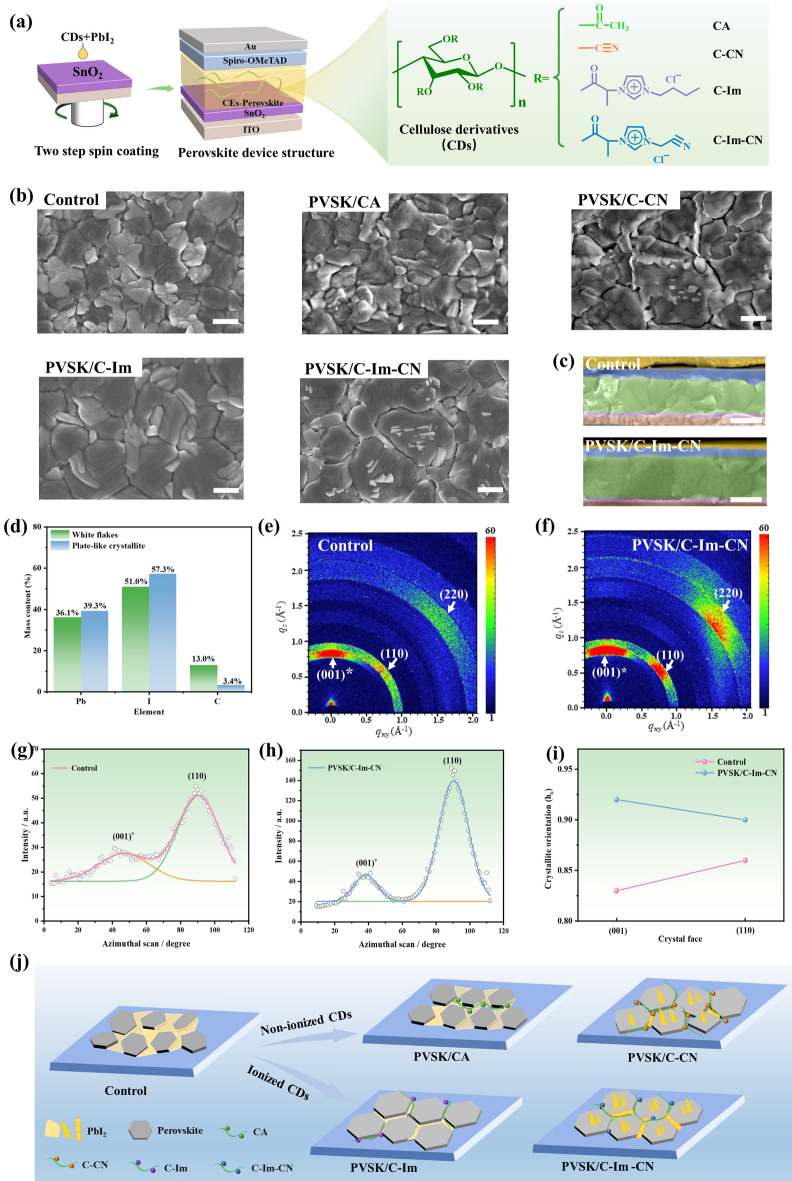
PSCs passivated with cellulose derivatives: **a** schematic illustration of PSCs passivated with cellulose derivatives; **b** SEM images of the surfaces of perovskites passivated with different cellulose derivatives (Scale bar: 500 nm); **c** cross-sectional SEM images of the control PSC and PSC passivated with C-Im-CN (PVSK/C-Im-CN) (Scale bar: 500 nm); **d** element contents of the plate-like crystallite and the white flake on the grain surface in PVSK/C-Im-CN; grazing incidence wide-angle X-ray scattering (GIWAXS) patterns of **e** the control PSC and **f** PVSK/C-Im-CN; Crystallite orientation fits to the characteristic peaks of PbI_2_ (orange line) and PVSK (green line) in **g** the control PSC and **h** PVSK/C-Im-CN; **i** crystallite orientation of the control PSC and PVSK/C-Im-CN at (110) and (001)* crystal faces; and **j** schematic illustration of perovskites passivated with different cellulose derivatives. (Note: (110) and (220) belong to PVSK; (001)* is assigned to PbI_2_)

In this work, we designed and synthesized a series of ionic and nonionic CDs as eco-friendly additives to participate in the perovskite (PVSK) growth (Fig. 1a). Among them, the ionic cellulose derivative C-Im-CN with cyano-imidazolium (Im-CN) cation and chloride anion provides multiple interaction sites, including the imidazolium cation, cyano group, hydroxyl group, carbonyl group, and chloride anion, which strongly interact with perovskite via electrostatic interactions, hydrogen-bonding interactions, and coordination interactions. Thus, the C-Im-CN markedly promotes the growth and directional orientation of perovskite grains, regulates excess PbI_2_, and optimizes the perovskite energy level. The resultant PSCs exhibit a high PCE of 24.71% with an improved long-term stability of over 3000 h.

## Experimental Section

### Materials

Microcrystalline cellulose (DP = 220) was dried in a vacuum oven at 60 °C for 48 h prior to use. The ionic liquid 1-allyl-3-methylimidazolium chloride (AmimCl) was synthesized in our laboratory. The water content in AmimCl determined by Karl Fischer method was less than 0.3 wt%. Tetrabutylphosphonium hydroxide ([P_4,4,4,4_]OH, 40 wt%), acrylonitrile (AN, 99%), 1-butylimidazole (98%), N-(2-cyanoethyl)-imidazole (98%), 2-chloropropionyl chloride (97%), and cellulose acetate (CA, DS = 2.5) were supplied by Beijing Innochem Science and Technology Co., Ltd. Tin oxide (SnO_2_, 15 wt%) and isopropanol (IPA, 99.7%) were purchased from Alfa Aesar. Lead iodide (PbI_2_, 99.9%), cesium iodide (CsI, 99.99%), formamidine iodide (FAI, 99.5%), methylammonium chloride (MACl, 99.5%), methylammonium bromide (MABr, 99.5%), Spiro-OMeTAD (99.8%), lithium bis(trifluoromethanesulfonyl)imide (Li-TFSI, 99.95%), 4-tert-butyl pyridine (TBP, 96%), acetonitrile (ACN, 99.5%), and 4-methoxyphenethylammonium (4-CH_3_O-PEAI, 99.5%) were purchased from Xi’an Polymer Light Technology in China. Chlorobenzene (CB, 99.8%), dimethylsulfoxide (DMSO, 99.7%), and dimethylformamide (DMF, 99.8%) were brought from Sigma-Aldrich. All chemicals were used without further purification.

### Methods

#### Synthesis of Cyanoethyl Cellulose (C–CN)

Microcrystalline cellulose (1.00 g, 6.17 mmol) was dispersed in [P_4,4,4,4_]OH aqueous solution (12.80 g). After stirring at 25 °C for 30 min, acrylonitrile (1.0 mL) was added drop by drop to the transparent cellulose solution. Then, the mixture solution was stirred at 0–10 °C for 4 h. The crude product was precipitated in a mixture of ethanol and water (1:1, v/v), filtered, washed more than three times, and finally dried in a vacuum oven for 24 h. The obtained C–CN has a DS of 1.25. ^1^H-NMR (400 MHz, DMSO-d_6_): *δ* 2.80–5.50 (m, 8H), 2.73 (s, 2H).

#### Synthesis of Cellulose 2-Chloropropionate (CCl)

Microcrystalline cellulose (1.00 g, 6.17 mmol) was dissolved in AmimCl (38.00 g) at 80 °C. Then, 2-chloropropionyl chloride (1.21 g, 9.50 mmol) was added into the cellulose/AmimCl solution under ice bath condition. After 10 min of stirring, the reaction system was transferred into an oil bath of 40 °C for 1 h. At the end of the reaction, the crude product was precipitated with ethanol, washed three times with ethanol, and finally dried in a vacuum oven at 60 °C. The obtained CCl has a DS of 1.85.

#### Synthesis of Cellulose 1-Butylimidazolium Chloride (C-Im)

The CCl (1.00 g, 2.70 mmol) was dissolved in DMF. Then, 1-butylimidazole (1.11 g, 8.90 mmol) was added into the CCl/DMF solution at 80 °C for 24 h. The product was precipitated in acetone and collected by filtration. After washing three times with acetone, the crude product was redissolved in dimethyl sulfoxide (DMSO), precipitated and washed with acetone. Finally, the product was filtered and dried under vacuum at 60 °C for 24 h to obtain C-Im with DS_Im_ = 1.35. ^1^H-NMR (400 MHz, DMSO-d_6_): *δ* 9.40 (s, 1H), 7.80 (s, 2H), 6.00–2.92 (m, 10H), 1.80 (s, 5H), 1.30 (s, 2H), 0.80 (s, 3H).

#### Synthesis of Cellulose Cyanomethylimidazolium Chloride (C-Im-CN)

The CCl (1.00 g, 2.70 mmol) was dissolved in DMF. Then, N-(2-cyanoethyl)-imidazole (1.2–3.2 g, 4.00–10.60 mmol) was added to the CCl/DMF solution at 80 °C for 24–48 h. The product was precipitated in acetone and collected by filtration. The crude product was washed with acetone three times. Finally, the product was dried under vacuum at 60 °C for 24 h before characterization. ^1^H-NMR (400 MHz, DMSO-d_6_): δ 9.40 (s, 1H), 7.80 (s, 2H), 6.00–2.92 (m, 10H), 1.82 (s, 3H).

### Device Fabrication

#### (FAPbI_3_)_1-x_(MAPbBr_3-y_Cl_y_)_x_ Device

The glass/ITO substrate was ultrasonically cleaned with deionized water, acetone, and isopropanol. Then, the ITO substrates were dried by the nitrogen gun and treated by UV-ozone for 10 min. The SnO_2_ solution (0.24 mL) was diluted with deionized water (1.35 mL), and then, it was dropped at glass/ITO substrate with a rate of 3000 rpm for 30 s. After that, the SnO_2_ ETL was achieved by annealing at 150 °C for 35 min (30%–40% RH). For the perovskite, PbI_2_ (1.65 mmol) was dissolved into DMF/DMSO (6.25:1, v/v) mixed solution, was spin-coated onto the SnO_2_ film at a rate of 1600 rpm for 30 s, and was annealed at 70 °C for 2 min. For perovskite/CDs, CDs were dissolved in PbI_2_ precursor solution with different concentrations ranging from 0.1 to 0.25 mg mL^−1^. The mixed amine solution was prepared by dissolving FAI (110 mg), MACl (11.5 mg), and MABr (11 mg) in IPA (1.5 mL), then spin-coated onto the PbI_2_ films at a rate of 2000 rpm for 25 s and annealed at 140 °C for 20 min in ambient air with a controlled humidity (30%–40% RH). For 2D/3D plus perovskite films, 100 μL of CH_3_O-PEAI isopropanol solution (3 mg mL^−1^) was spin-coated on the 3D perovskite films surface at a rate of 4000 rpm for 30 s, then annealed at 100 °C for 10 min. For the HTL, the Spiro-OMeTAD solution was obtained by mixing Spiro-OMeTAD (72.3 mg), TBP (28.8 μL), Li-TFSI/ACN solution (35 μL, 260 mg Li-TFSI in 1 mL ACN), and chlorobenzene (1 mL). After that, it was spin-coated on perovskite films at a rate of 5000 rpm for 40 s. Then, the devices were kept in a desiccator for 20 h. Finally, 90 nm of Au was evaporated on the HTL surface as the electrode.

### Measurements

^1^H-NMR spectra were measured on a Bruker AV-400 NMR spectrometer (Bruker, USA) with 16 scans at room temperature. Samples were dissolved in DMSO-d_6_, and 2–3 drops of trifluoroacetic acid-d_1_ were added to transfer active hydrogen to the low field. FTIR spectra were measured by using Thermo Nicolet 6700 Fourier transform infrared spectrometer (Thermo Fisher, USA). The wavenumber range was 650–4000 cm^−1^, the resolution was 4 cm^−1^, the scanning mode was a reflection mode, and the scans number was 64. UV–vis transmission spectra were obtained on Perkin-Elmer Lambda 35 UV–vis spectrometer (Perkin-Elmer, USA). The scan range was 400–800 nm with a scan interval of 1 nm. XPS spectra were obtained on X-ray photoelectron spectrometer ESCALab250 i-XL (Thermo Fisher Scientific, USA). UPS data were obtained from Kratons AXIS ULTRA DLD, and the HeI (21.22 eV) excitation line was employed. X-ray diffractometer (XRD) patterns were recorded by using a D/MAX-2500 X-ray diffractometer (Rigaku Denki, Japan). The X-ray radiation was Cu K_*α*_ with a wavelength of 1.5406 Å generated at 40 kV and 200 mA. Grazing incidence wide-angle X-ray scattering (GIWAXS) patterns were acquired at XEUSS WAXS/SAXS system (Xenocs, France). The sample-to-detector distance was 132 mm, the wavelength of the incident X-rays was 1.5418 Å, and the angle of incidence was 0.5°. Scanning electron microscopy (SEM) and energy dispersive spectrometer (EDS) images were observed by using a JEOL JSM-8020 field-emission scanning electron microscope (JEOL, Japan) at 5–10 kV. Photoluminescence (PL) spectra were obtained from FLS980 fluorescence spectrometer, excited at 485 nm and measured at 786 nm. Raman spectra were investigated by LabRAM HR Evolution with an excitation light source of 485 nm (HORIBA, France). *J-V* curves were measured by using Keithley 2420 under AM 1.5G illumination (Newport 94043A, USA). The device area was 0.04 cm^2^. EQE spectra were measured using Newport 300 W Xenon Light Source (Newport IQE 200, USA). The EIS was obtained by Zennium (Zahner, Germany). Static contact angle was measured by a droplet size analyzer DSA-100 (Krüss, Germany).

## Results and Discussion

### Perovskite Passivated with Cellulose Derivatives

Natural cellulose is rich in hydroxyl groups; thus in theory, the structure and properties can be easily modified after a chemical modification [41–44]. Via a Michael addition reaction, cyanoethyl cellulose (C–CN) was homogeneously synthesized in [P_4,4,4,4_]OH aqueous solution (Fig. S1a). Via a two-step derivatization process, the butylimidazolium cation (Im) and cyanomethylimidazolium cation (Im-CN) were introduced on the cellulose chain (C) to obtain cationic CDs of C-Im and C-Im-CN, respectively (Fig. S1b, c). In ^1^H-NMR spectrum of C–CN, the peak at 2.7 ppm is attributed to the methylene protons of -CH_2_CN, and the peaks at 2.6–5.5 ppm are assigned to the protons of cellulose skeleton and the methylene protons of -O-CH_2_- in cyanoethyl group (Fig. S2). In ^1^H-NMR spectra of C-Im and C-Im-CN, the peaks at 9.4 and 7.8 ppm are attributed to the protons of the imidazolium cation, the peaks at 2.9–6.0 ppm are the protons of cellulose skeleton and the methylene/methine protons of –CH_2_–N– and –CH–N–, and the peaks at 0.8–1.8 ppm are assigned to the methylene and methyl protons of the butylimidazolium and cyanomethylimidazolium cations. The degree of substitution (DS) of C–CN, C-Im, and C-Im-CN is calculated as 1.25, 1.35, and 0.63, respectively, according to ^1^H-NMR spectra. C–CN, C-Im, C-Im-CN, and commercial cellulose diacetate (CA, DS = 2.50) were severally added into the mixed PbI_2_/CsI solution to participate in the perovskite crystallization (Fig. 1a). By a two-step spin coating process, the perovskite films passivated with four CDs were obtained.

In the control sample, the grain size of perovskite is about 420 nm, and there is a large amount of PbI_2_ at the grain boundaries (Figs. 1b and S3). After using the above four CDs, the microstructure of the passivated perovskites is significantly changed (Figs. 1b, c and S4). In the perovskite passivated with CA (PVSK/CA), the grain size increases to 609 nm and the PbI_2_ at the grain boundaries decreases, but the grain surface becomes extremely bumpy. In the PVSK/C-CN, the grain size increases significantly to 860 nm, and the PbI_2_ at the grain boundaries remarkedly reduces. The white flakes appear on the grain surface. But, each gain is composed of small crystal particles and has anomalous shape. Moreover, there are many wide vacancies at the grain boundaries. In the PVSK/C-Im, the grain size increases evidently to 751 nm, and the grain surface is flat and homogeneous. In the PVSK/C-Im-CN, the grain size increases markedly to 849 nm, and the grain surface is flat and homogeneous also. A large number of white flakes appear on the grain surface; meanwhile, new plate-like crystallites are distributed vertically in the local domains (Figs. 1b and S4). Even if there are white flakes on the surface, the PVSK/C-Im-CN film has the lowest roughness (Fig. S5). The micro-EDS images confirm that the white flakes and plate-like crystallites are mainly composed of Pb and I elements. The Pb/I molar ratio is about 1:2.3, which is close to that of PbI_2_ and is different from that of the perovskite crystal (dark region) (Figs. 1d and S6–S8) [45]. So, the white flakes and plate-like crystallites should be PbI_2_ [46, 47]. The addition of C-Im-CN not only promotes the enlargement of perovskite grains, but also regulates the distribution of excess PbI_2_, remarkably reducing the defects at grain boundaries [16, 48–50].

All C-Im-CN samples with different DS values exhibit a promotion effect on perovskite growth and a distribution management of excess PbI_2_ (Fig. S9). In addition, the C-Im-CN concentration has an obvious impact on the PbI_2_ regulation. For example, C-Im-CN with DS = 0.63 indicates the strongest effect at a concentration of 0.15 mg mL^−1^ (Fig. S10). In addition, the control perovskite crystals have random orientation and poor interfacial connection (Fig. 1c). In contrast, perovskite grains in PVSK/C-Im-CN exhibit completely vertical alignment, large size running through the whole perovskite layer, and tight connection (Figs. 1c and S11).

In XRD patterns of the perovskites passivated with four CDs, the main peaks are the (110), (220), and (310) crystal planes of the perovskite, and the (001) crystal plane of PbI_2_ (Fig. S12) [51]. There is no new peak. Due to the large size of long polymer chain, CDs are just optimizing the perovskite crystallization rather than entering the internal lattice of perovskite [39]. Meanwhile, after using CDs, the (001) plane of PbI_2_ dramatically decreases, especially in PVSK/C-Im-CN. This suggests that the crystals contain only a small amount of PbI_2_, owing to the management effect of C-Im-CN passivator [52], agreeing with the SEM and AFM images in which PbI_2_ is transferred to the grain surface to form smaller crystals. Besides, in grazing incidence wide-angle X-ray scattering diffraction (GIWAXS), scatter factors (*q*_z_) of 0.85, 0.93, and 2.04 Å^−1^ in Bragg reflections correspond to (001) plane of PbI_2_, (110) and (220) planes of perovskite, respectively (Fig. 1e, f). The integrated intensity of GIWASX data along *q*_z_ indicates that the relative intensity of the (110) diffraction peak of the PVSK/C-Im-CN is markedly higher than that of the control (Figs. S13 and S14), confirming that the perovskite grains have the better orientation after the C-Im-CN treatment [53–55]. By data fitting, the full width at half-maximum intensity (FWHM) of azimuthal plot was used to calculate the crystallite orientation (*h*_c_) (Fig. 1 g, h). The *h*_c_ values of (001) and (110) crystal faces in the control are 0.83 and 0.86, respectively, while the *h*_c_ values of (001) and (110) crystal faces in PVSK/C-Im-CN films increase to 0.92 and 0.90, directly proving that the addition of C-Im-CN effectively improves the orientation degree of PbI_2_ and perovskite crystals (Fig. 1i and Table S1). It is consistent with the cross-sectional SEM results and XRD patterns. Therefore, C-Im-CN promotes perovskite crystallization most effectively and conducts the enrichment of excess PbI_2_ on the grain surface and in local regions, eliminating the perovskite grain boundary defects.

All four CDs can facilitate the growth of perovskite grains, especially C–CN, C-Im, and C-Im-CN (Fig. 1j). But, when the nonionic CDs of CA and C–CN were used, the surface of the perovskite grains deteriorated, which led to poor carrier transportation. In contrast, when the ionic cellulose derivative C-Im-CN was used, the perovskite grains not only had a large size, which was twice as much as that of the control sample, but also had flat and homogeneous surface. Moreover, the perovskite grains in PVSK/C-Im-CN exhibited regular orientation and tight connection. In addition, the C-Im-CN conducted the transfer of excess PbI_2_ to the surface of perovskite grains or the enrichment of excess PbI_2_ in local domains, inhibiting further formation of the defects. Thus, C-Im-CN hopefully elevates the PCEs of PSCs.

### Mechanism of Cellulose Derivatives Passivating Perovskite

**Fig. 2:**
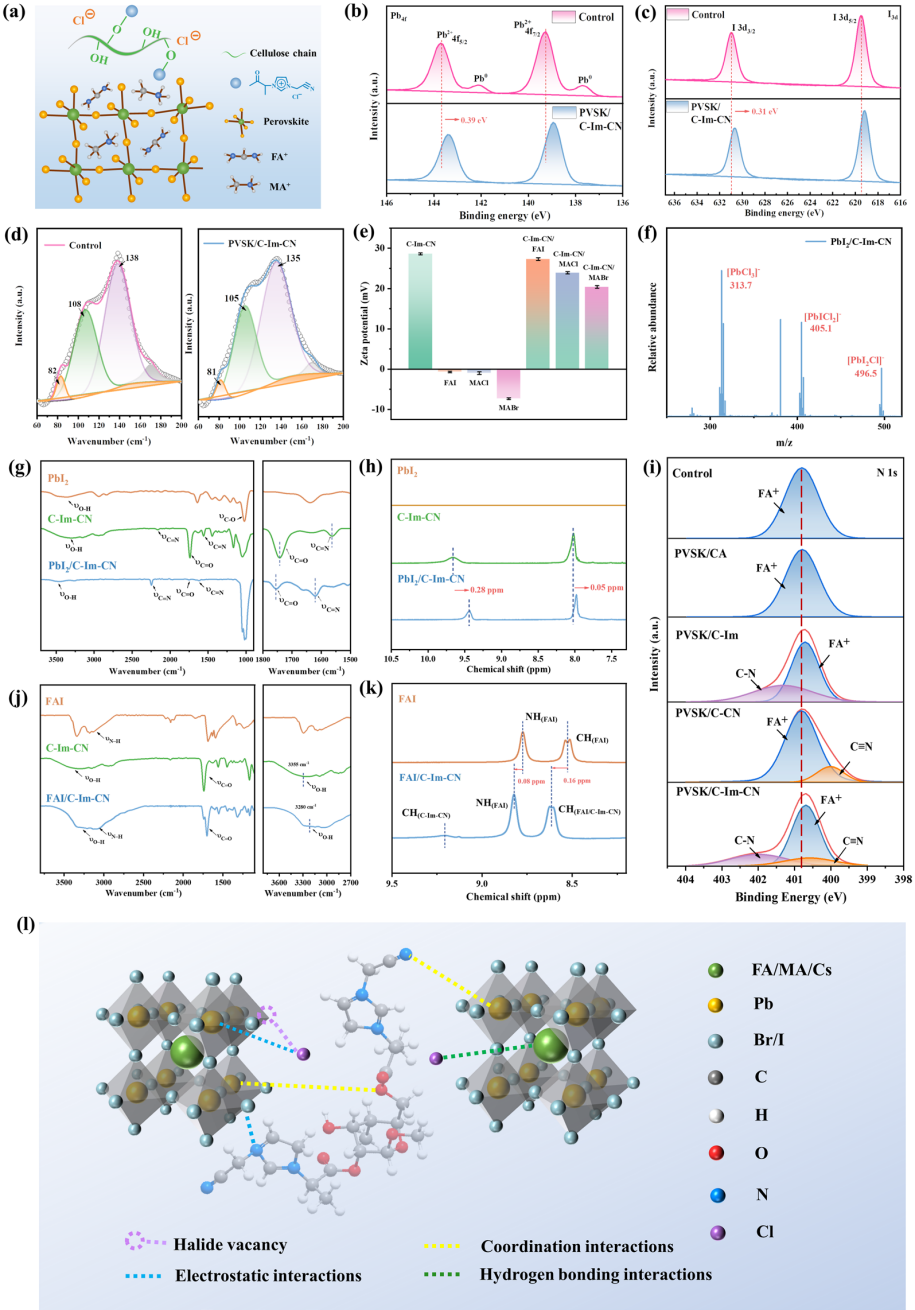
Mechanism of cellulose derivatives passivating perovskite: **a** chemical structure of C-Im-CN and perovskite; **b** Pb 4*f* high-resolution XPS spectra of control and PVSK/C-Im-CN; **c** I 3*d* high-resolution XPS spectra of control and PVSK/C-Im-CN; **d** Raman spectra of control and PVSK/C-Im-CN; **e** zeta potentials of C-Im-CN, FAI, MACl, MABr, C-Im-CN/FAI, C-Im-CN/MACl, and C-Im-CN/MABr; **f** mass spectra of PbI_2_/C-Im-CN solution; **g** FTIR spectra of PbI_2_, C-Im-CN, and PbI_2_/C-Im-CN; **h** ^1^H-NMR spectra of PbI_2_, C-Im-CN, and PbI_2_/C-Im-CN; **i** N 1*s* high-resolution XPS spectra of control and perovskites passivated with cellulose derivatives; **j** FTIR spectra of FAI, C-Im-CN, and FAI/C-Im-CN; **k** ^1^H-NMR spectra of FAI and FAI/C-Im-CN; and (**I**) schematic illustration of the interactions between C-Im-CN and perovskite

C-Im-CN has several functional groups, including Cl^−^ anion, cyano group, carbonyl group, hydroxyl group, and imidazolium cation (Fig. 2a). They strongly interact with Pb^2+^ cation, FA^+^ cation, MA^+^ cation, I^−^ anion, and Br^−^ anion in perovskite, via electrostatic interaction, coordination interaction, and hydrogen-bonding interaction. Compared with those in the control perovskite, the Pb 4*f* peak of PVSK/C-Im-CN shifts 0.39 eV, and the I 3*d* peak shifts 0.31 eV toward the low binding energy region (Fig. 2b, c). Meanwhile, the uncoordinated Pb^0^ in the control perovskite totally disappears after using C-Im-CN, suggesting that the C-Im-CN inhibits the defects in perovskite [56]. In Raman spectra, the peaks at 82, 108, and 138 cm^−1^ in perovskite shift to lower frequency region after the addition of C-Im-CN (Fig. 2d). These three peaks represent the second phonon mode of [PbI_6_]^4−^, the asymmetric stretching vibration of I-Pb-I, and the oscillation mode of [PbI_6_]^4−^ octahedron, respectively [45]. These above phenomena indicate that there are strong interactions between C-Im-CN and perovskite [11, 57, 58]. After the addition of C-Im-CN solution into the ammonium salt (FAI, MACl, and MABr) solutions, the ζ-potential changes from negative to positive (Fig. 2e), indicating that C-Im-CN forms electrostatic interactions with the anions and cations of perovskite. After mixing PbI_2_ solution with C-Im-CN solution, [PbI_2_Cl]^−^, [PbICl_2_]^−^, and [PbCl_3_]^−^ complexes form, further demonstrating a strong coordination interaction between the PbI_2_ and the Cl^−^ anion in C-Im-CN (Fig. 2f). Moreover, the stretching vibration peak of C=O shifts from 1742 to 1755 cm^−1^, the C≡N peak shifts from 2163 to 2251 cm^−1^, and the C=N peak shifts from 1564 to 1620 cm^−1^ (Fig. 2g). FTIR results confirm that the C=O and C≡N in C-Im-CN form strong coordination interactions with Pb^2+^, and the imidazolium cation in C-Im-CN forms electrostatic interactions with I^−^ anion. XPS results show the similar conclusion (Fig. S15). In addition, the hydrogen on the imidazolium cation shifts toward the higher field after mixing the PbI_2_ solution with C-Im-CN solution (Fig. 2h), demonstrating that the formation of complexes between the Cl^−^ anion and Pb^2+^ ions weakens the hydrogen-bonding interaction between the Cl^−^ anion and the imidazolium cation. Besides, compared with the control perovskite, the N 1*s* peaks of PVSK/C-Im and PVSK/C-Im-CN shift toward lower binding energies, while the N 1*s* peaks of PVSK/CA and PVSK/C–CN show no shift, indicating that the Cl^−^ anions form electrostatic and/or hydrogen-bonding interactions with FA^+^ cations in perovskites (Fig. 2i). After mixing FAI solution with C-Im-CN solution, the O–H stretching vibration peak shifts from 3355 to 3280 cm^−1^, and the N–H stretching vibration peak shifts from 3180 to 3071 cm^−1^, demonstrating that FAI forms multiple hydrogen-bonding interactions with C-Im-CN (Fig. 2j). Furthermore, the amine hydrogen of FAI at 8.99 ppm shifts to 9.07 ppm which demonstrates that the Cl^−^ anion forms a hydrogen-bonding interaction with the FA^+^ ion (Fig. 2k). Therefore, the Cl^−^ anion, cyano group, and carbonyl group in C-Im-CN form coordination interactions with Pb^2+^ ion; the Cl^−^ anion, hydroxyl group, cyano group, and carbonyl group in C-Im-CN form hydrogen-bonding interactions with FA^+^ ion and halogen anion in perovskite; and the imidazolium cation and Cl^−^ anion in C-Im-CN form electrostatic interactions with Pb^2+^ ion, FA^+^ ion and halogen anion in perovskite (Fig. 2l). As a result, C-Im-CN markedly promotes the grain growth and directional orientation of perovskite, suppresses the ion migration, vacancies and boundary defects, as well as conducts the transfer of PbI_2_.

The detailed mechanism is deduced as follows: In the first step, C-Im-CN was dissolved in PbI_2_/DMF/DMSO solution. The C≡N groups anchored with [PbI_6_]^4−^ via coordination interaction to form the nucleation sites [59]. Because the C≡N groups regularly arranged along the cellulose chain, the resultant PbI_2_ crystals exhibited an improved orientation. XRD results showed that, after adding C-Im-CN, the FWHM of PbI_2_ at 13.0° decreased (Fig. S16), and GIWAXS patterns exhibited that the (001) diffraction ring of PbI_2_ became narrower (Fig. S17). In addition, the C-Im-CN with a plenty of interaction sites had multi-interactions with Pb^2+^ and I^−^, so many intermediate phases of PbI_2_ formed. As a result, the mesoporous PbI_2_ with a small size was obtained (Fig. S18). This small, mesoporous, and oriented PbI_2_ benefited the subsequent growth and orientation of perovskite grains [60]. Besides, Cl^−^ anion interacted with Pb^2+^ to form an intermediate phase [PbI_2_Cl_x_]^x−^, which was more conducive to the subsequent reaction of PbI_2_ and ammonium salts. Then, the FAI/MACl/MABr/IPA solution was spin-coated onto the PbI_2_ film. During the annealing process, some halide vacancy appeared during the solvent evaporation. Because C-Im-CN chain surrounded the crystals, it provided Cl^−^ anion to compensate for the halide vacancy via electrostatic interaction and/or hydrogen-bonding interaction between Cl^−^ and FA^+^/MA^+^/Pb^2+^ [61, 62]. It also provided imidazolium cation to further stabilize the position of halide anions (I^−^ and Br^−^) via electrostatic interaction and/or hydrogen-bonding interaction, reducing the migration of halide anions. Thus, the defects in perovskite reduced, the perovskite grains became perfect, and the grain size increased. Based on the multi-interaction sites and long-chain effect, C-Im-CN significantly improves the quality and grain orientation of perovskite.

### Device Performance

**Fig. 3:**
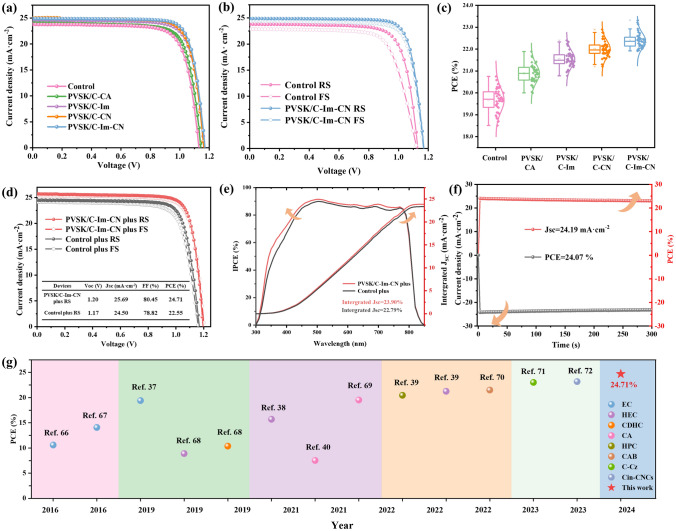
Photovoltaic performance of control and perovskites passivated with cellulose derivatives: **a** *J–V* curves of PSCs; **b** *J–V* curves of PSCs measured at reverse and forward scans; **c** distribution graphs of PCEs (Box plots and normal distribution curves are displayed next to the data points. Each case is collected from 40 independent devices.); **d** *J–V* curves of PSCs of PVSK/C-Im-CN plus measured at reverse and forward scans; **e** IPCE and the integrated *J*_*SC*_ of control plus and PVSK/C-Im-CN plus; **f** maximal steady-state photocurrent and stabilized PCE of PVSK/C-Im-CN plus; and **g** photovoltaic efficiency of PSCs based on different cellulose additives in recent years

We prepared devices with the n-i-p structure of ITO/SnO_2_/perovskites/spiro-OMeTAD/Au (Fig. 1a). The control device delivers a PCE of 20.58%, open-circuit voltage (*V*_OC_) of 1.13 V, short-circuit current density (*J*_SC_) of 23.77 mA cm^−2^, and fill factor (*FF*) of 76.72%, under standard simulated AM 1.5G illumination in reverse scan (from 1.2 V to −0.2 V) (Fig. 3a and Table S2). All four CDs have a positive impact on the performance of the PSCs. In particular, the PSC passivated with C-Im-CN yields a PCE of 23.35% with a *V*_OC_ of 1.17 V, *J*_SC_ of 24.90 mA cm^−2^, and *FF* of 80.16%. The increase of *V*_OC_ is attributed to the reduction of non-radiative recombination and the optimization of energy level alignment. The enhanced *J*_SC_ and *FF* demonstrate that PVSK/C-Im-CN has fewer defects [63, 64]. The charge rapidly transports in PVSK/C-Im-CN, and the recombination of carriers is suppressed at the perovskite/HTL interface. The PSC of PVSK/C-Im-CN exhibits an extremely low hysteresis index (HI) of 2.75% which is much lower than that of the control device (9.52%) (Fig. 3b and Table S3), indicating that the addition of C-Im-CN inhibits the ion migration. For the repeat testing, 40 devices were fabricated under the same conditions, and the average PCEs obviously increased with the addition of CDs (Figs. 3c and S19). In addition, the PSCs were biased at the initial maximum power point voltage for 300 s (Fig. S20). The PCEs of the control PSC and PVSK/C-Im-CN PSC remain 20.19% and 22.89%, respectively. The PVSK/C-Im-CN PSC exhibits a higher and more stable power output (SPO) compared with the control device. In PVSK/C-Im-CN PSC, the outstanding efficiency, low HI value, and excellent operational stability are ascribed to the superior effect of C-Im-CN on the passivation of perovskite defects, which is most favorable for crystal growth.

To further promote the perovskite/HTL interface, we added *p*-methoxyphenethylammonium iodide (CH_3_O-PEAI) in the post-treatment process of perovskite films (denoted as control plus and C-Im-CN plus) [65]. The characteristic peaks of PVSK/C-Im-CN crystals treated with CH_3_O-PEAI had high intensity and crystallinity also (Fig. S21). After the treatment, the PCE of the PVSK/C-Im-CN PSC was enhanced to 24.71%, with *V*_OC_ of 1.20 V, *J*_SC_ of 25.69 mA cm^−2^, and *FF* of 80.45% (Fig. 3d), because CH_3_O-PEAI assisted in the hole extraction and reduced the interfacial charge accumulation. The incident photon-to-charge conversion efficiency (IPCE) and the integrated *J*_SC_ of the PSC of C-Im-CN plus showed evidence of small deviations from *J-V* curves (Fig. 3e). The C-Im-CN plus device was held at a bias of 1.02 V for 300 s, and the *J*_SC_ was stabilized at 24.19 mA cm^−2^, leading to a SPO of 24.07% (Fig. 3f).

The effect of different concentrations and DS of C-Im-CN on the PCEs was systematically investigated. With the increase of C-Im-CN concentration, the device efficiency firstly increased and then decreased (Fig. S22 and Table S5). The PCE reached the highest value at a concentration of 0.15 mg mL^−1^. Excess C-Im-CN would occupy the grain boundary space, leading to the accumulation of vacancies and defects (Fig. S7). The DS of C-Im-CN had an obvious effect on the PCEs also (Fig. S23 and Table S6). C-Im-CN with DS = 0.35 gave a limited improvement in *V*_OC_ and *FF*. C-Im-CN with DS = 2.66 suppressed the *FF*, probably because the few OH groups were unfavorable to the passivation of defects owing to the weak hydrogen-bonding acidity. C-Im-CN with DS = 0.63 had an optimal effect on the improvement of the PCE. We compared the champion parameters of the C-Im-CN plus device with the PSCs passivated with other cellulose-based additives reported in the past year (Fig. 3g and Table S7) [37–40, 66–72]. Obviously, the C-Im-CN plus device had the best PCE.

**Fig. 4:**
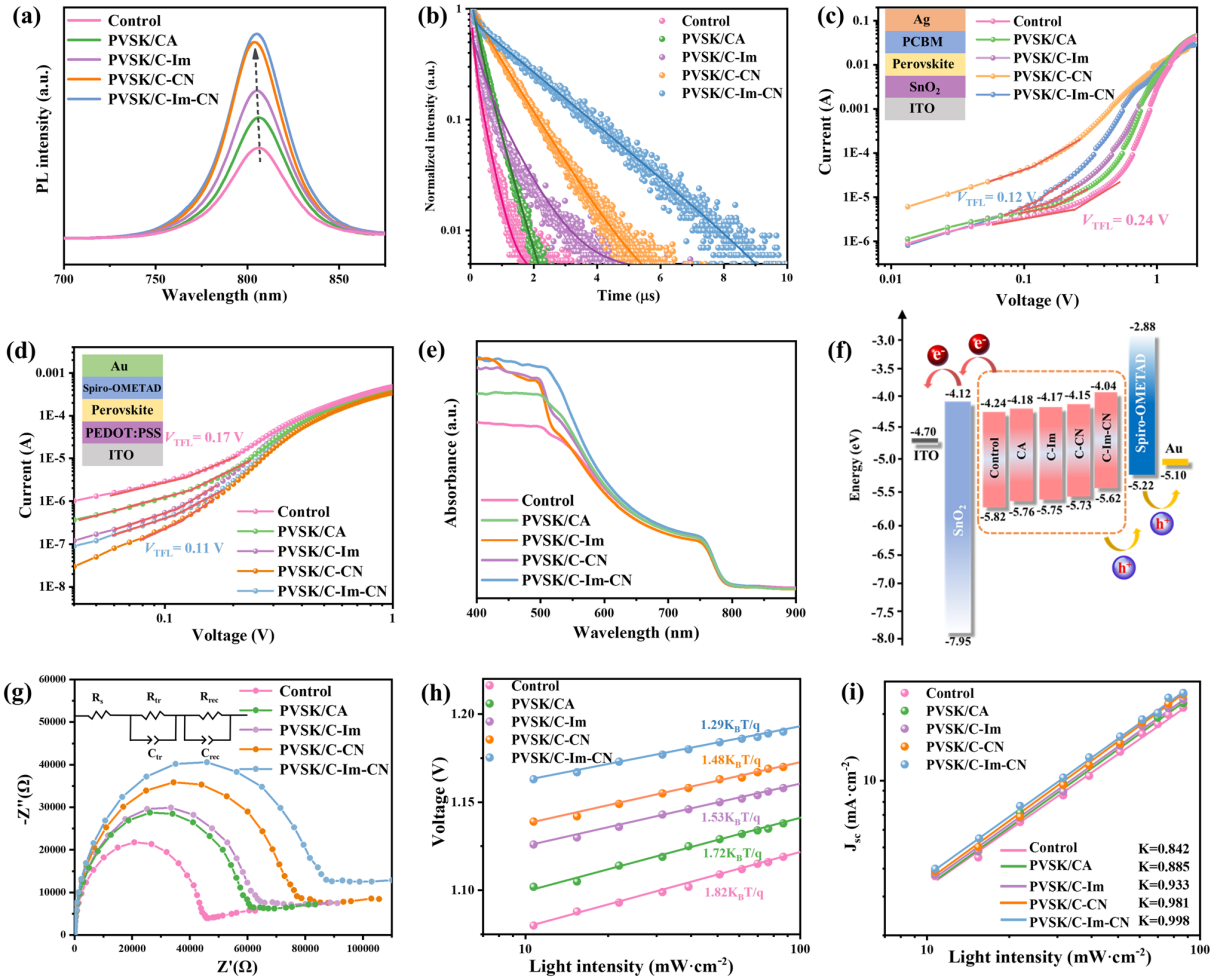
Characterization of perovskites with or without cellulose derivatives: **a** steady-state PL spectra of perovskites with or without cellulose derivatives; **b** PL lifetime spectra of perovskites; **c** dark *J–V* curves for the electron only devices with the structure of ITO/SnO_2_/perovskite/PCBM/Ag; **d** dark *J–V* curves for the hole only devices with the structure of ITO/PEDOT:PSS/perovskite/Spiro-OMETAD; **e** UV–vis absorption spectra of perovskites; **f** energy level illustration of the main functional layers in PSCs; **g** Nyquist plots of PSCs; and **h** *V*_OC_ and **i** *J*_SC_ response under different light intensities

The PL intensity of the perovskites significantly increased after the addition of four CDs, and the emission peaks shifted to the lower wavelengths compared with that of the control (Fig. 4a). These phenomena claim that the CDs effectively reduce the defects of perovskite. The PVSK/C-Im-CN not only exhibited the highest PL intensity, but also maximized the carrier lifetime (τ) for 937 ns, which was 3–4 times higher than that of the control (Fig. 4b and Table S8), proving the evident improvement of perovskite quality and the reduction of defects. Subsequently, following the equation *N*_t_ = 2ε_0_ε_r_*V*_TFL_/e*L*^2^ (*N*_t_: defect density of states; *V*_TFL_: defect filling limiting voltage), the defect density of perovskites was quantified by space charge limited current (SCLC) measurement (Figs. 4c, d and S24, S25) [73, 74]. The dark-state *J–V* curves for the electron-only devices showed that the *V*_TFL_ and *N*_t_ of PVSK/C-Im-CN were 0.12 V and 1.0 × 10^15^ cm^−3^, respectively, which were much lower than those of the control (0.24 V, 2.0 × 10^15^ cm^−3^). The dark-state *J-V* curves for the hole-only devices showed that the *V*_TFL_ and *N*_t_ of PVSK/C-Im-CN were 0.11 V and 0.92 × 10^15^ cm^−3^, respectively, which were much lower than that of the control (0.17 V, 1.4 × 10^15^ cm^−3^) also. All the above results demonstrate that the addition of C-Im-CN can effectively reduce the defect density and promote the perfection of perovskite crystals.

The reduction of defects facilitates the extraction and transport of carriers. The addition of four CDs improved the light absorption capability of perovskite in 400–760 nm region, which was benefit to improve the *J*_sc_ in varying degree (Fig. 4e). The addition of four CDs had a negligible effect on the absorption of 800 nm light, suggesting that the band gaps remained the same and were 1.58 eV. According to the UPS result, the conduction band energy level (*E*_c_) and the valence band energy level (*E*_v_) of the control were −4.24 eV and −5.82 eV, respectively, while those of perovskite/C-Im-CN film were −4.04 eV and −5.76 eV (Figs. S26 and S27). The corresponding energy band alignment is depicted in Fig. 4f. The higher *E*_v_ of the perovskite/C-Im-CN film facilitated a more efficient hole extraction, significantly reducing the charge accumulation and recombination at the interface between HTL and perovskite [75]. Therefore, the well-matched energy level should be profit to enhance FF and PCE of the devices.

The effect of the CDs on the interfacial charge transfer and recombination was analyzed by electrochemical impedance spectroscopy (EIS) (Fig. 4g) [76, 77]. Compared with the control device, the devices with CDs showed an obvious decrease in arc in the high-frequency region, implying a decrease in the charge transfer resistance (*R*_tr_), and a gradual increase in arc in the low-frequency region, representing an evident increase in the charge complexation resistance (*R*_rec_). We tested the variation curves of *V*_OC_ and *J*_SC_ with light intensity in order to investigate the internal carrier transport mechanism. The relationship between *V*_OC_ and light intensity was in accordance with the natural logarithmic relationship, in which the PVSK/C-Im-CN corresponded to the smallest slope, so it had the strongest ability to suppress the carrier recombination caused by defects and reduce the energy loss (Fig. 4h) [78]. In addition, there was a clear linear relationship between *J*_SC_ and light intensity, and a larger slope facilitated charge extraction [75]. The C-Im-CN had the highest slope of 0.998 (Fig. 4i). Therefore, the addition of CDs enhances the carrier extraction and transport and suppresses the non-radiative recombination.

The above results prove that the four CDs, especially C-Im-CN, promote the perovskite crystallization, reduce the defects, enhance the carrier extraction and transport, reduce ion migration and non-radiative recombination, and optimize the energy level of perovskite, thus facilitating the charge enrichment at the electrodes.

### Stability of PSCs

**Fig. 5:**
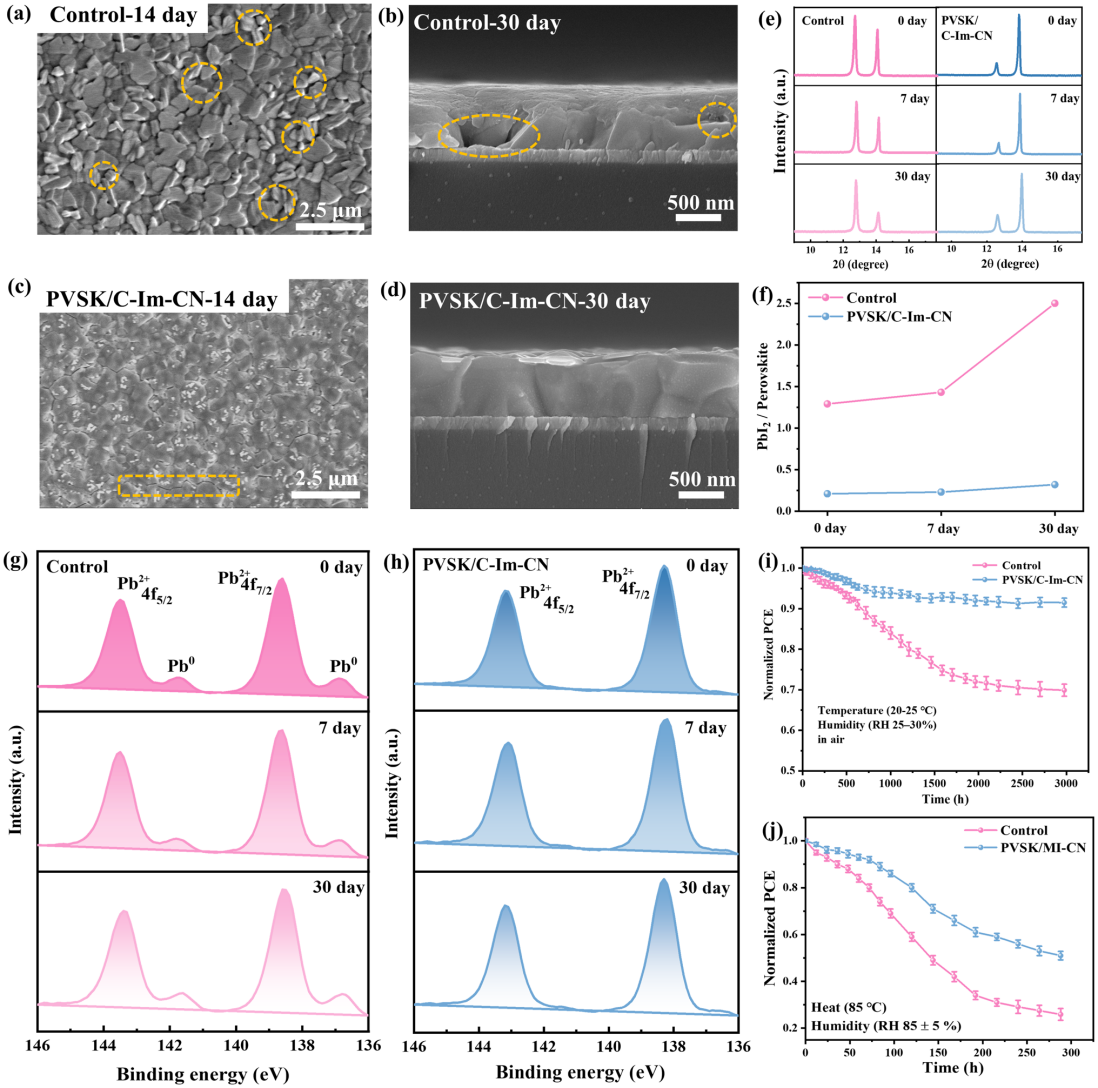
Device stability test of the control and PVSK/C-Im-CN: **a** and **b** SEM images of the surface and cross-section of the control under air environment after 14-30 days (RH = 25%-30%, T = 20-25 °C); **c** and **d** SEM images of the surface and cross-section of the PVSK/C-Im-CN under air environment after 14-30 days (RH = 25%-30%, T = 20-25 °C); **e** XRD spectra of the control and PVSK/C-Im-CN under air environment for different days (RH = 25%-30%, T = 20-25 °C); **f** Ratio of PbI_2_/perovskite crystal peak of the control and PVSK/C-Im-CN; **g** and **h** Pb 4*f* high-resolution XPS spectra of the control and PVSK/C-Im-CN before and after the aging period under air environment (RH = 25%-30%, T = 20-25 °C); **i** Stability of the control and PVSK/C-Im-CN devices under 3,000 h (RH = 25%-30%, T = 20-25 °C); **j** Stability of the control and the PVSK/C-Im-CN devices under high humidity condition of 300 h (RH = 85±5%, T = 85 °C)

After leaving the control perovskite in air for 14–30 days, SEM images showed that the bulk perovskite crystal cracked into smaller grains, voids appeared, and the film surface became bumpy (Fig. 5a). A cross-section of the perovskite crystal showed many obvious voids and cracks (Fig. 5b). The deterioration of perovskite crystal originated from the escape of organic cations, which not only further accelerated ionic migration, but also released more cation and halide vacancies [79]. By contrast, the PVSK/C-Im-CN had a less surface degradation, and most of domains maintained the original crystal structure with few defects only at the grain boundaries (Fig. 5c). Similarly, the crystal in the cross-section remained intact without the vacancies or holes (Fig. 5d). The same effect was also observed in perovskites stored in a high humidity environment. The control perovskite was severely broken, while the PVSK/C-Im-CN had only a few vacancies (Fig. S28). These phenomena were attributed to the multiple interactions between perovskite and C-Im-CN. Moreover, unlike small molecules with individual functional groups, C-Im-CN had a long chain and plenty of interaction sites. The cyano group, hydroxyl group, carbonyl group, and chloride anion in C-Im-CN strongly interacted with the FA^+^ and MA^+^ cations via the strong hydrogen-bonding and electrostatic interactions, effectively inhibiting the cation escape. Numerous hydroxyl groups and imidazolium cations in C-Im-CN formed hydrogen-bonding and electrostatic interactions with I^−^/Br^−^ anions in perovskite, which effectively reduced the halide vacancy defects and further inhibited the generation of metallic Pb (Pb^0^).

In order to further clarify the protection effect of C-Im-CN on perovskite, we monitored the perovskite films with XPS and XRD measurements before and after the aging period. As the erosion of moisture and oxygen, the intensity of PbI_2_ diffraction peak of the control dramatically increased, and the ratio of the characteristic peaks of PbI_2_ and perovskite crystals increased from 1.29 to 2.50 (Fig. 5e, f). In PVSK/C-Im-CN, the intensity of PbI_2_ diffraction peak increased slightly, and the ratio of PbI_2_/perovskite only changed from 0.21 to 0.32, proving that the addition of C-Im-CN was beneficial to the stability of the perovskite crystal in a long-term period.

In the control perovskite, the excess PbI_2_ was significantly decomposed into Pb^0^ (Fig. 5g), while the PVSK/C-Im-CN had no obvious Pb^0^ peaks in the XPS curves even when it was placed until 30 days (Fig. 5h). It is worth to be noted that the excess PbI_2_ was still well preserved at the grain surfaces in PVSK/C-Im-CN (Fig. 5c). These phenomena suggest that the addition of C-Im-CN stabilized the excess PbI_2_ also, thus reducing Pb^0^ generation, which further demonstrated that the C-Im-CN with multiple interaction sites was favorable for the long-term stability of the devices.

After operated for 3000 h in a conventional air atmosphere, the PCE of the PVSK/C-Im-CN still retained 91.3% of the original value, which was much higher than the 69.9% of the control device (Fig. 5i). When devices were operated at a high humidity environment for 300 h, the PCE of PVSK/C-Im-CN devices remained 81.0%, while the control was only 64.1% (Fig. S28c). After 300-h treatment at 85 °C under a high RH of 85 ± 5%, the PCE of the PVSK/C-Im-CN remained 51.0%, while the efficiency of the control decreased to 25.9% (Fig. 5j). The C-Im-CN thus effectively protected the devices under air and extreme environments owing to its multiple interaction sites and polymer skeleton.

## Conclusion

To achieve high PCEs of perovskite solar cells, perfect crystal structure and less defect are essential. A series of CDs with different substituents have been designed and used to manage the crystallization engineering of perovskite. Among them, the cationic cellulose derivative C-Im-CN can effectively promote grain growth and directional orientation of perovskite crystal, meanwhile suppress defect formation and decrease grain boundaries. Because in C-Im-CN, the imidazolium cation, cyano group, hydroxyl group, carbonyl group, and chloride anion strongly interact with halogen anion, Pb^2+^ cation, and FA^+^/MA^+^/Cs^+^ cations in perovskite via electrostatic interactions, hydrogen-bonding interactions, and coordination interactions. Moreover, the addition of C-Im-CN enables the transfer of excess PbI_2_ to the surface of perovskite grains, or the aggregation of excess PbI_2_ in local domains, further inhibiting the  formation of defects. Photovoltaic and photoluminescence performance demonstrates that C-Im-CN promotes the perfection of perovskite crystals, reduces the defects, enhances the carrier extraction and transport, optimizes the energy level of perovskite, and reduces ion migration and non-radiative recombination. As a result, all the photovoltaic parameters of the PSCs including *J*_SC_, *V*_OC_, and *FF* are enhanced, and a highest PCE of 24.71% is achieved. In addition, the C-Im-CN with multiple interaction sites and polymer skeleton is beneficial to improve the stability of PSCs. After operated for 3000 h in a conventional air atmosphere, the unencapsulated PSCs maintain above 91.3% of their initial efficiencies. Therefore, cellulose materials provide a promising strategy for constructing the PSCs with high-performance and long-term stability.

## Supplementary Information

Below is the link to the electronic supplementary material.

Supplementary file1 (PDF 2319 kb)
