# Supplementary material for: Manipulating Crystal Growth and Secondary Phase PbI2 to Enable Efficient and Stable Perovskite Solar Cells with Natural Additives
Source: Nanomicro Lett. 2024 Apr 29;16:183. doi: 10.1007/s40820-024-01400-w (PMC11058175; doi:10.1007/s40820-024-01400-w)
Supplement: Supplementary file 1 — Supplementary file1 (PDF 2319 kb) [file 40820_2024_1400_MOESM1_ESM.pdf]

Supporting Information for

Manipulating Crystal Growth and Secondary Phase PbI_2 to Enable Efficient and Stable Perovskite Solar Cells with Natural Additives

Yirong Wang[1,2], Yaohui Cheng[3], Chunchun Yin[1], Jinming Zhang[1,*], Jingxuan You[1,2], Jizheng Wang[1,2,*], Jinfeng Wang[4], Jun Zhang[1,2]

[1] Institute of Chemistry, Chinese Academy of Sciences (CAS), Beijing 100190, P. R. China

[2] University of Chinese Academy of Sciences, Beijing 100049, P. R. China

[3] Nanjing University, Nanjing 210023, P. R. China

[4] Wuhan Textile University, Wuhan 430200, P. R. China

*Corresponding authors. E-mail: zhjm@iccas.ac.cn (Jinming Zhang); jizheng@iccas.ac.cn (Jizheng Wang)

Supplementary Figures and Tables

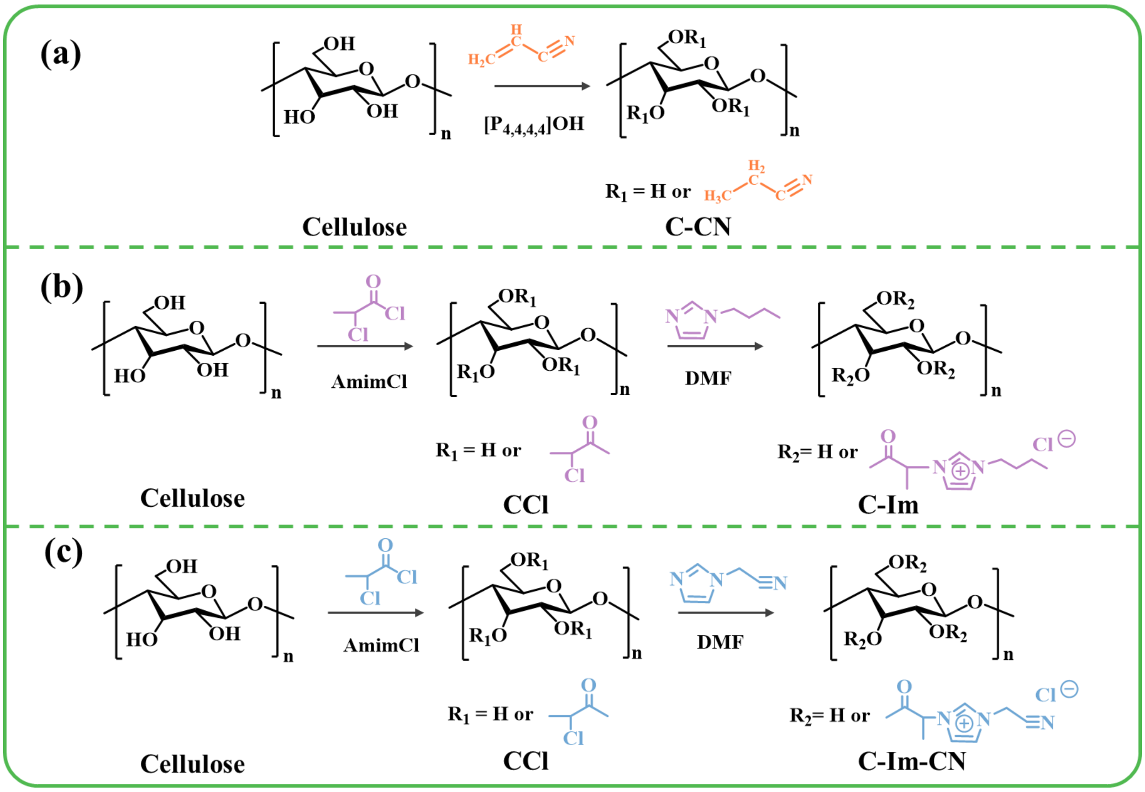

Fig. S1 Synthesis process of cellulose derivatives of (**a**) C-CN, (**b**) C-Im and (**c**) C-Im-CN

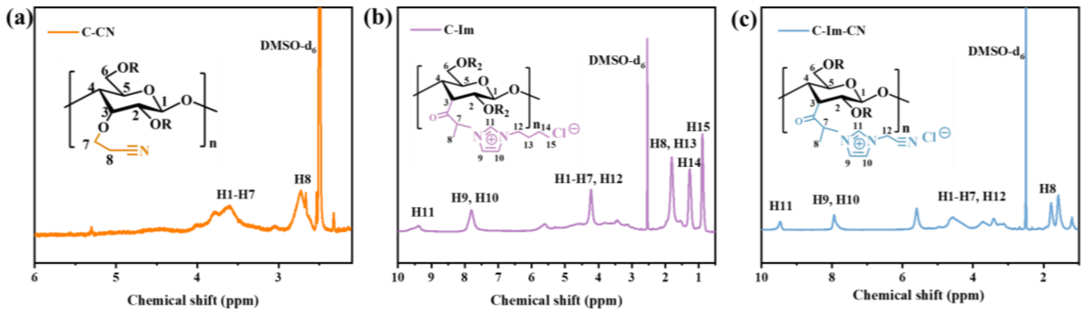

Fig. S2 ^{1}H-NMR spectra of cellulose derivatives of (**a**) C-CN, (**b**) C-Im and (**c**) C-Im-CN

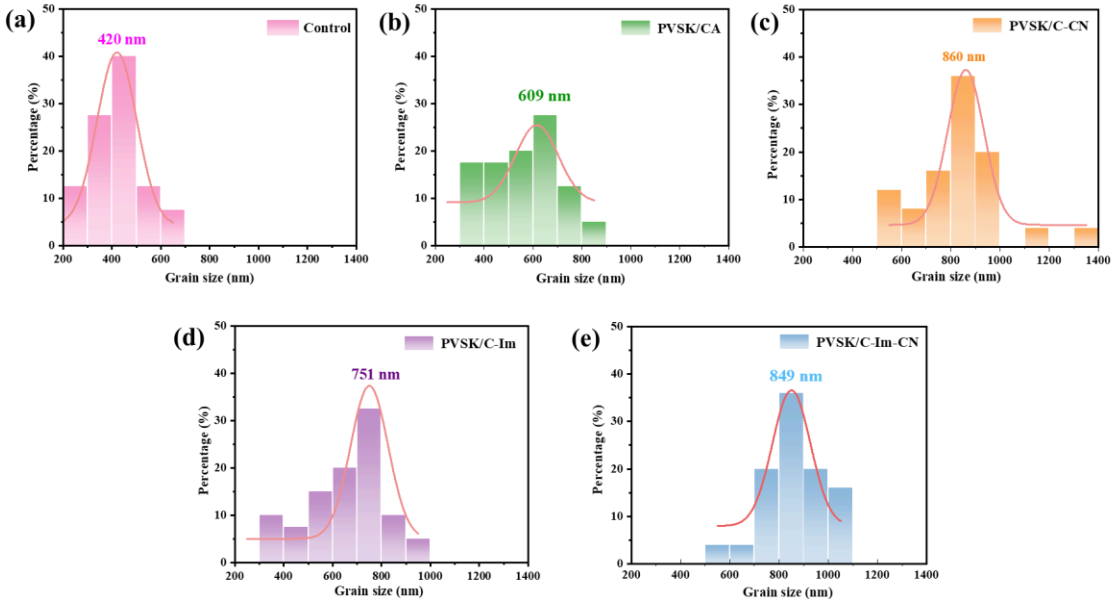

Fig. S3 Statistic grain size of the control and perovskites passivated with different cellulose derivatives

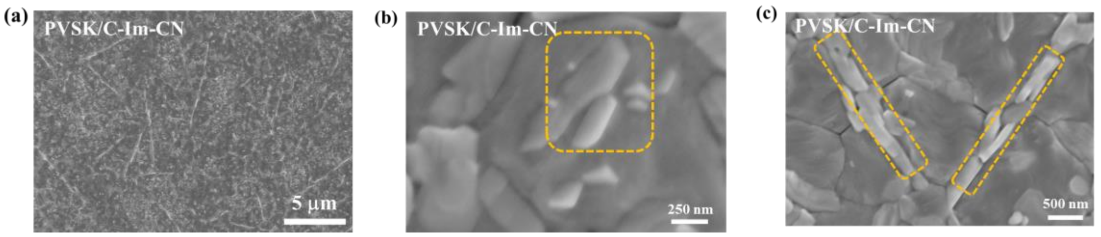

Fig. S4 (**a-c**) Top-view SEM images of PVSK/C-Im-CN film

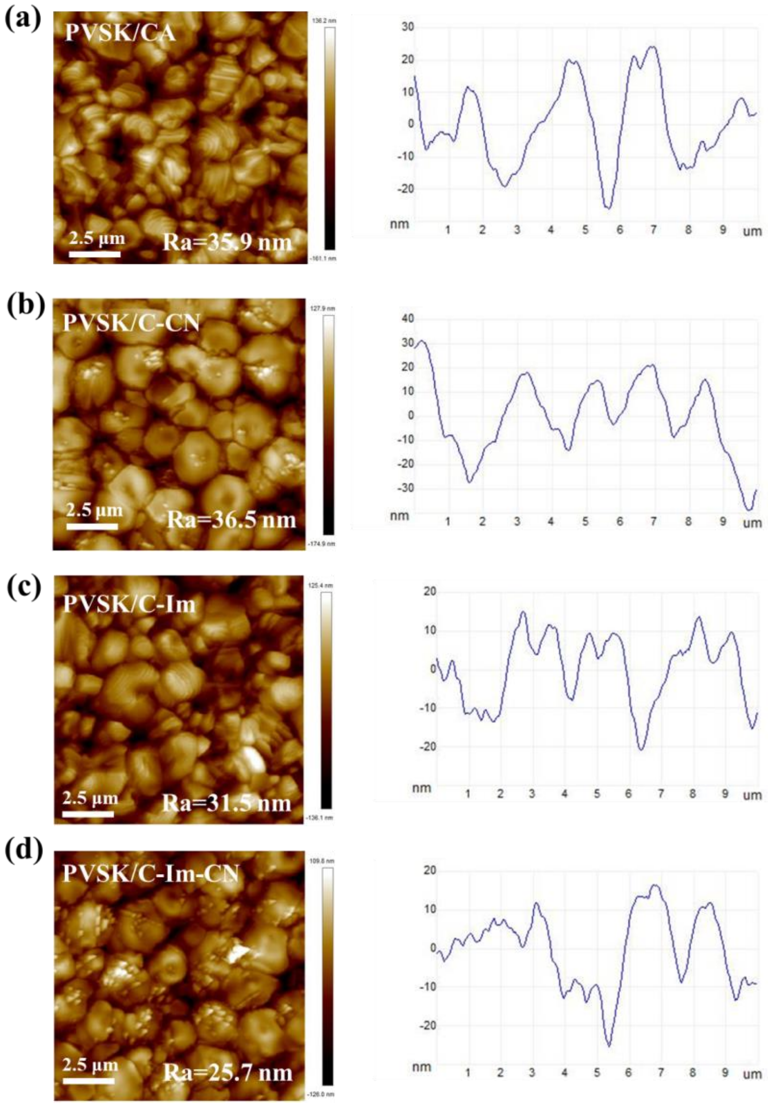

Fig. S5 AFM images and height distribution curves of the perovskite films treated with cellulose derivatives

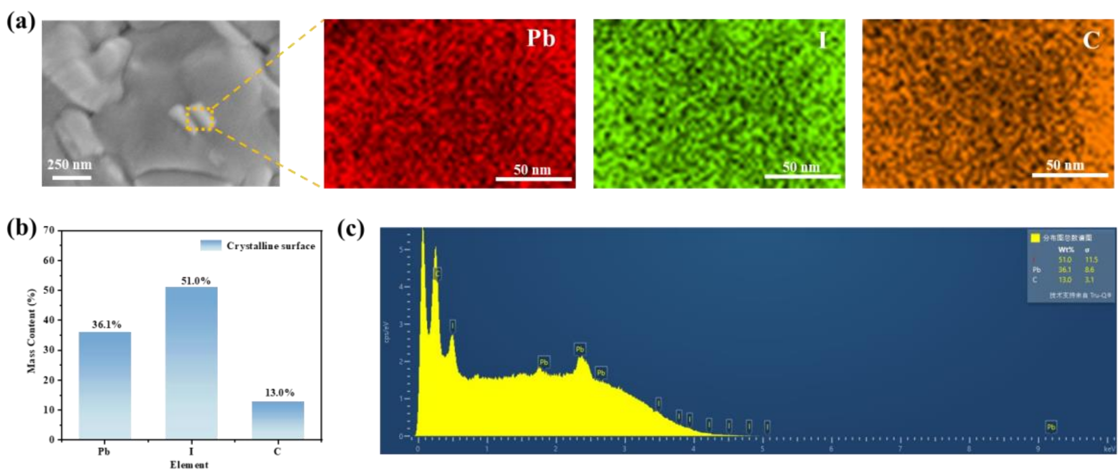

Fig. S6 TOP-view SEM image, EDS maps and element content map of the “white flakes” on grain surface of perovskite passivated with C-Im-CN

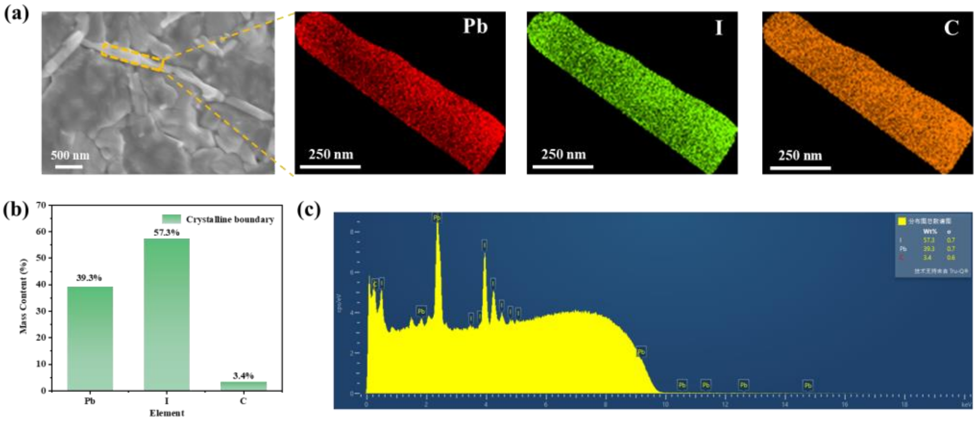

Fig. S7 TOP-view SEM image, EDS maps and element content map of the "plate-like crystallite" at grain boundary of perovskite passivated with C-Im-CN

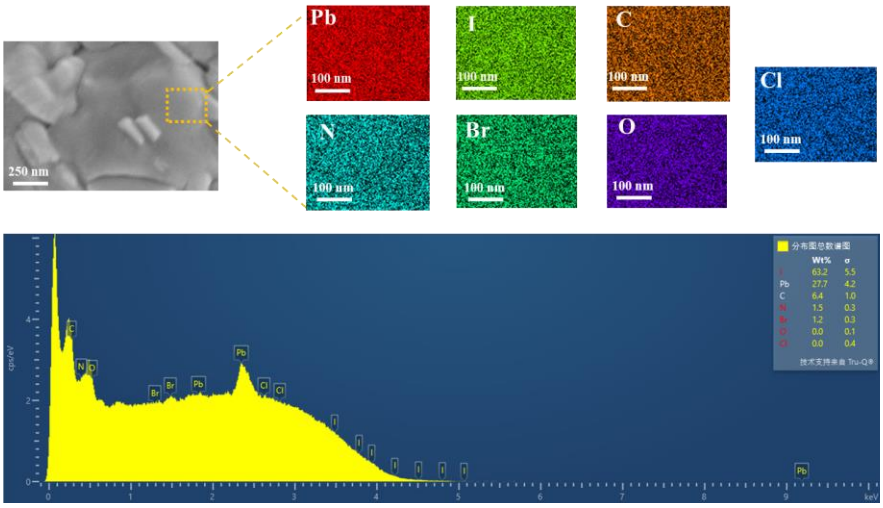

Fig. S8 TOP-view SEM image, EDS maps and element content map of the dark regions in the PVSK/C-Im-CN film

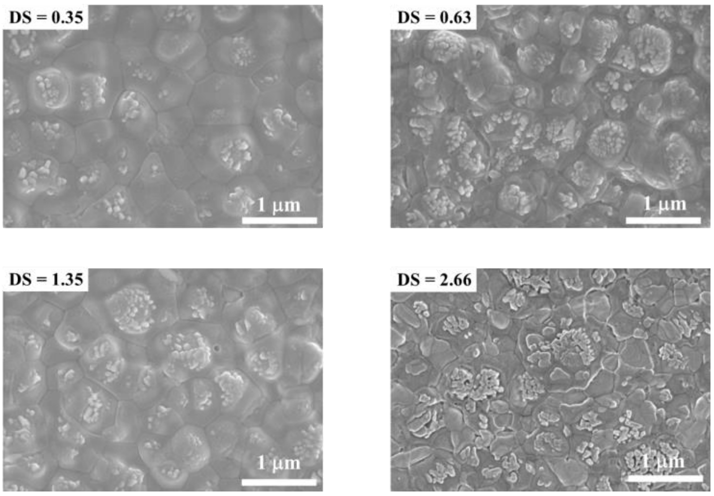

Fig. S9 Top-view SEM images of PVSK/C-Im-CN films with different DS values of C-Im-CN

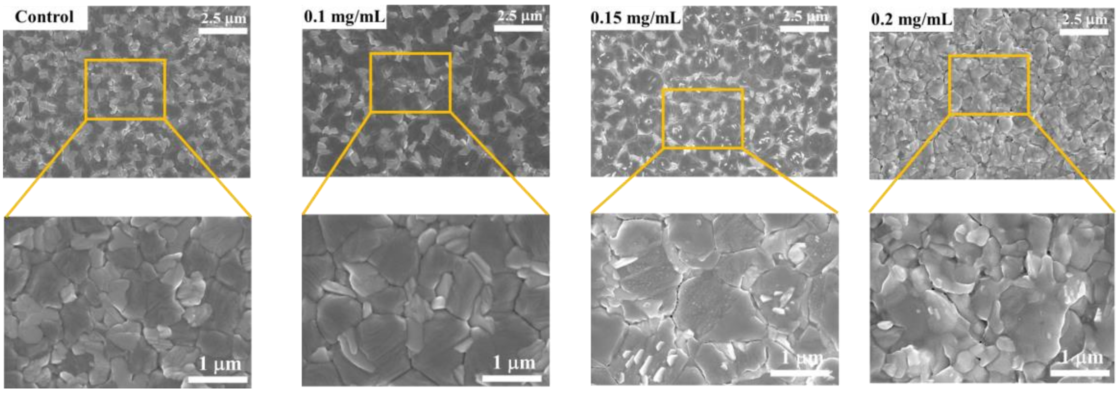

Fig. S10 Top-view SEM images of the control and PVSK/C-Im-CN films with different concentration of C-Im-CN

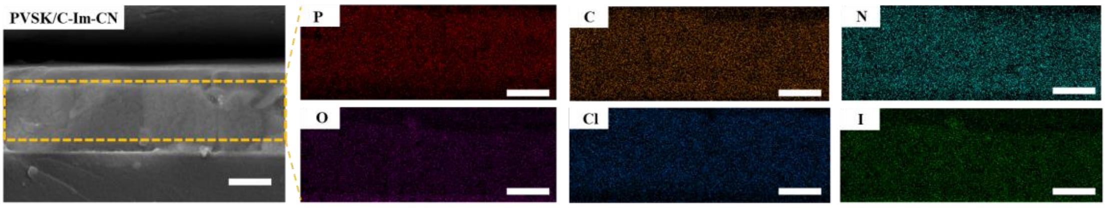

Fig. S11 Cross-sectional SEM image and EDS mapping of PVSK/C-Im-CN film (Scale bar: 500 nm)

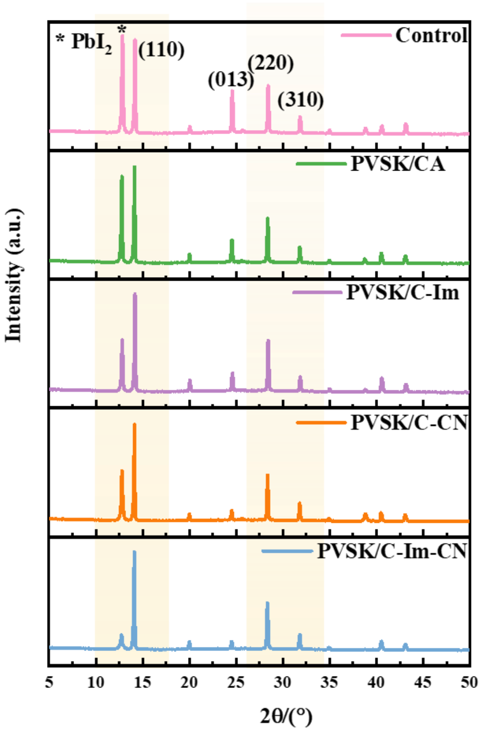

Fig. S12 XRD spectra of perovskites passivated with cellulose derivatives

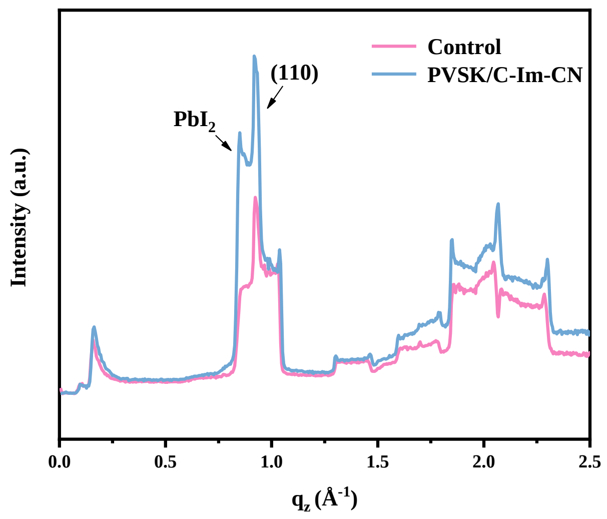

Fig. S13 Intensity vs. scatter factor (q_z) profiles of the control and PVSK/C-Im-CN taken from 2D GIWAXS patterns

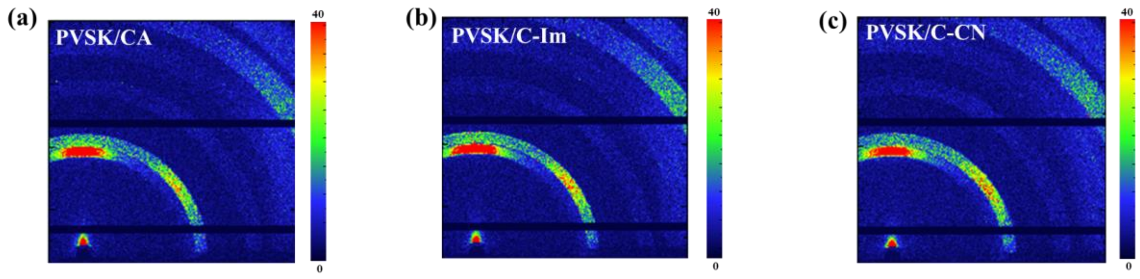

Fig. S14 GIWAXS patterns of the perovskite films passivated with different cellulose derivatives

Table S1 Data fit of crystal orientation from 2D GIWAXS patterns of the control and PVSK/C-Im-CN

Sample	FWHM (001)	h_{c1}	FWHM (110)	h_{c2}
**Control**	29.85	0.83	24.40	0.86
**PVSK/C-Im-CN**	14.09	0.92	17.57	0.90

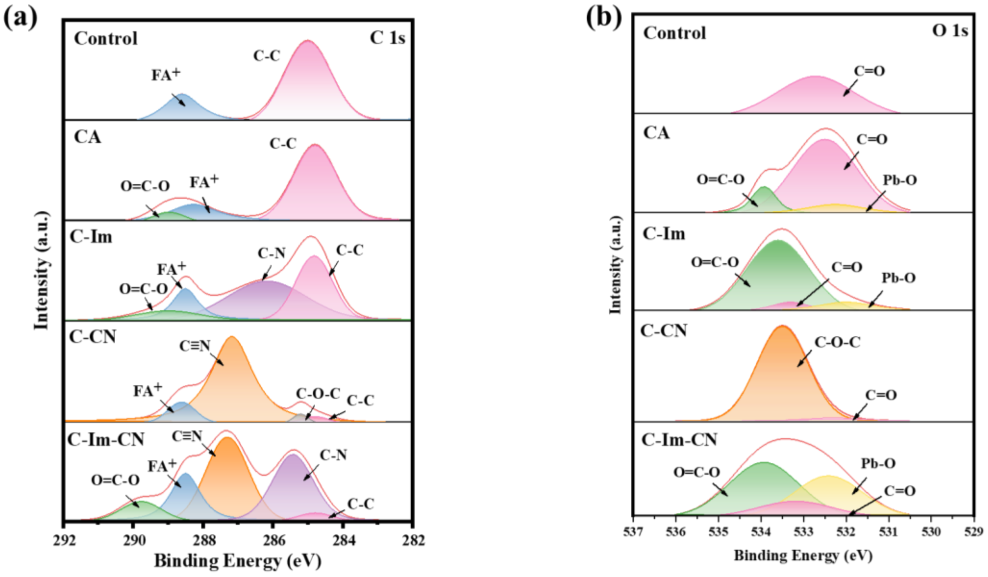

Fig. S15 (**a**) C 1s and (**b**) O 1s high-resolution XPS spectra of control and perovskites passivated with cellulose derivatives

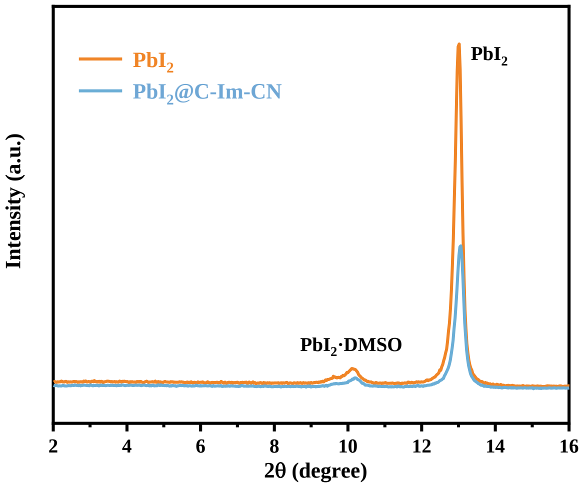

Fig. S16 XRD curves of PbI_2 and PbI_2@C-Im-CN films

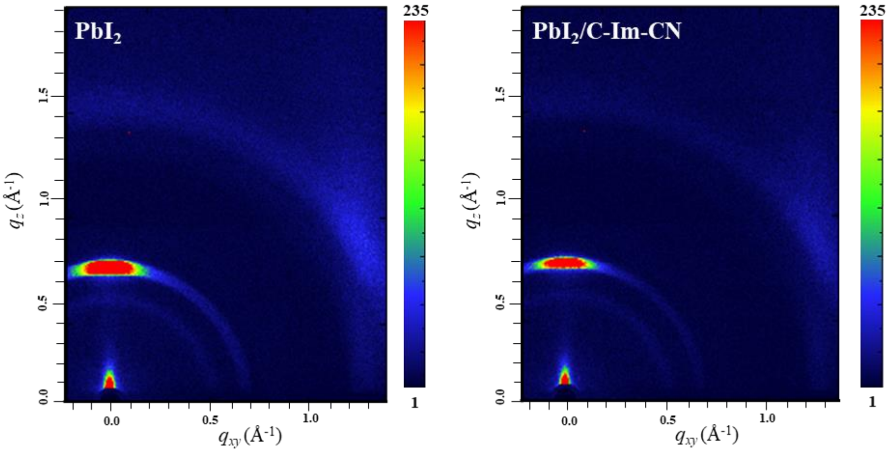

Fig. S17 GIWAXS patterns of (**a**) PbI_2 and (**b**) PbI_2/C-Im-CN films

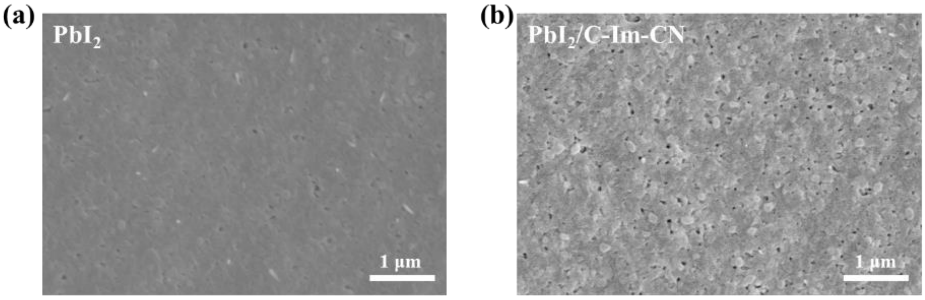

Fig. S18 TOP-view SEM images of (**a**) PbI_2 and (**b**) PbI_2/C-Im-CN films

Table S2 Efficiency and detailed performance parameters of the control and perovskites with different cellulose derivatives

Devices	V_{oc} (V)	J_{sc} (mA·cm^{-2})	FF (%)	PCE (%)
**Control**	1.13	23.77	76.72	20.58
**PVSK/CA**	1.14	24.05	77.19	21.20
**PVSK/C-Im**	1.16	24.67	77.93	22.42
**PVSK/C-CN**	1.16	25.07	78.52	22.88
**PVSK/C-Im-CN**	1.17	24.90	80.16	23.35

Table S3 Efficiency, HI index and detailed performance parameters of reverse and forward of the control and PVSK/C-Im-CN

Devices	V_{oc} (V)	J_{sc} (mA·cm^{-2})	FF (%)	PCE (%)	HI index (%)
**Control-RS**	1.13	23.77	76.72	20.58	9.52
**Control-FS**	1.12	22.87	72.57	18.62	
**PVSK/C-Im-CN-RS**	1.17	24.90	80.16	23.35	2.75
**PVSK/C-Im-CN-FS**	1.17	24.31	79.55	22.57	

Table S4 Efficiency, HI index and detailed performance parameters of reverse and forward of the control plus and PVSK/C-Im-CN plus.

Devices	V_{oc} (V)	J_{sc} (mA·cm^{-2})	FF (%)	PCE (%)	HI index (%)
**Control plus-RS**	1.17	24.50	78.82	22.55	5.14
**Control plus-FS**	1.16	24.14	76.35	21.39	
**PVSK/C-Im-CN plus-RS**	1.20	25.69	80.45	24.71	2.59
**PVSK/C-Im-CN plus-FS**	1.20	25.09	79.50	24.06	

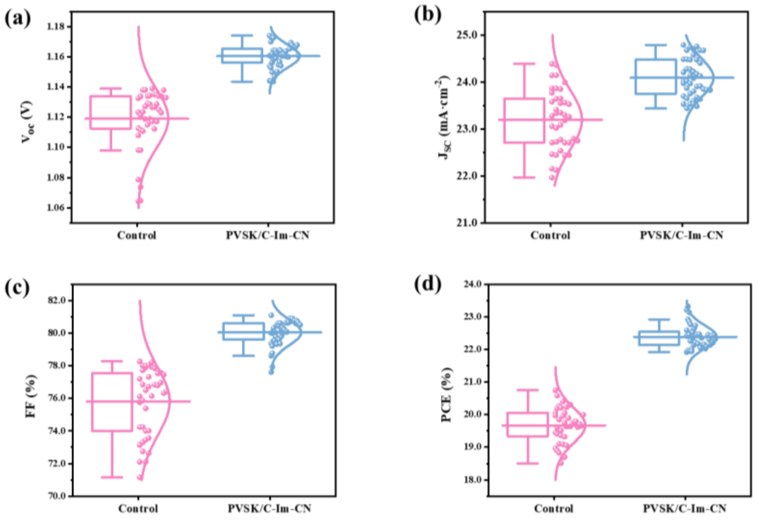

Fig. S19 Statistical distribution of experimental J-V parameters with PVSK/C-Im-CN devices. (**a**) Voc; (**b**) Jsc; (**c**) FF; (**d**) PCE

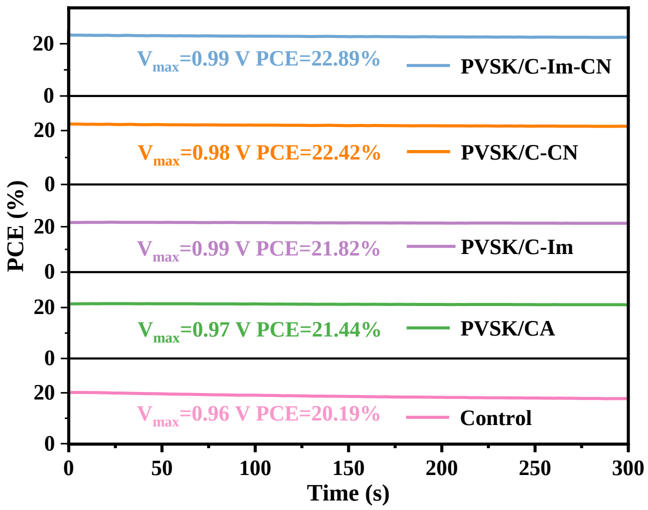

Fig. S20 Steady-state PCE versus time for the champion devices of the control and PVSK/cellulose derivatives measured at maximum power point (without CH_3O-PEAI)

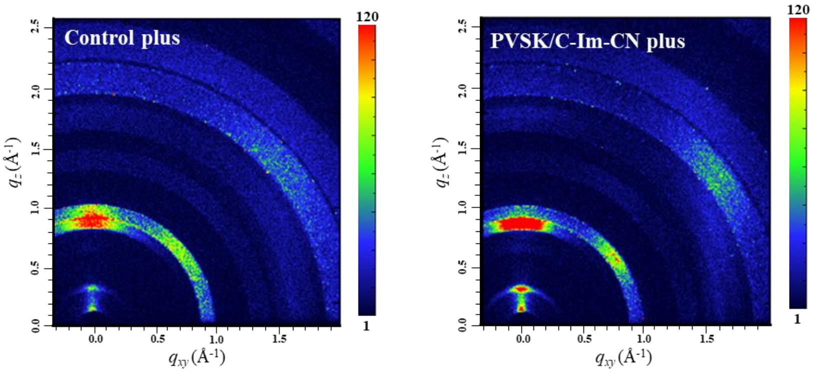

Fig. S21 GIWAXS patterns of the control and PVSK/C-Im-CN treated with CH_3O-PEAI

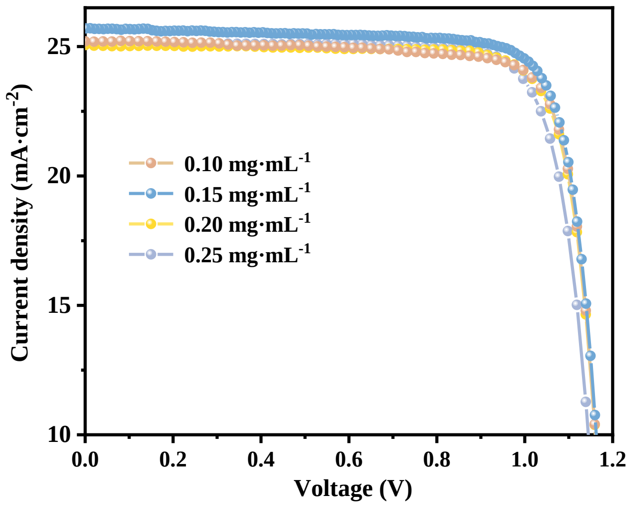

Fig. S22 J-V curves of PVSK/C-Im-CN devices fabricated by using C-Im-CN/DMF/DMSO with different concentrations of C-Im-CN

Table S5 Efficiency and detailed performance parameters of PVSK/C-Im-CN devices fabricated by using C-Im-CN/DMF/DMSO with different concentrations of C-Im-CN

C-Im-CN	V_{oc} (V)	J_{sc} (mA·cm^{-2})	FF (%)	PCE (%)
**0.10 mg·mL^{-1}**	1.19	25.04	80.75	24.15
**0.15 mg·mL^{-1}**	1.20	25.69	80.45	24.71
**0.20 mg·mL^{-1}**	1.19	25.23	80.45	24.23
**0.25 mg·mL^{-1}**	1.18	25.12	79.67	23.66

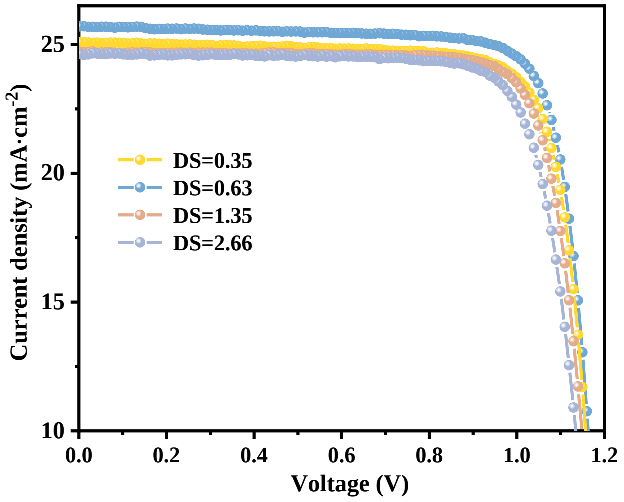

Fig. S23 J-V curves of PVSK/C-Im-CN devices with different DS of C-Im-CN

Table S6 Efficiency and detailed performance parameters of PVSK/C-Im-CN devices with different DS of C-Im-CN

C-Im-CN (DS)	V_{oc} (V)	J_{sc} (mA·cm^{-2})	FF (%)	PCE (%)
**0.35**	1.20	25.09	79.74	24.02
**0.63**	1.20	25.69	80.45	24.71
**1.35**	1.19	24.72	79.69	23.47
**2.66**	1.18	24.63	77.87	22.67

Table S7 Efficiency and detailed performance parameters of PSCs based on different cellulose additives in recent years [S1–S11]

Type	Voc (V)	Jsc (mA·cm^{-2})	FF (%)	PCE (%)	Year and Ref.
EC	0.90	18.62	63.00	10.60	2016 [1]
EC	0.99	21.18	67.20	14.08	2016 [2]
EC	1.10	22.89	77.10	19.41	2019 [3]
HEC	0.89	16.12	62.00	8.90	2019 [4]
CDHC	0.96	17.73	61.00	10.38	2019 [4]
HEC	1.12	17.90	78.50	15.70	2021 [5]
CA	1.08	10.88	64.00	7.52	2021 [6]
CA	1.11	23.05	76.33	19.53	2021 [7]
HPC	1.15	22.75	78.39	20.46	2022 [8]
HEC	1.16	23.02	79.74	21.26	2022 [8]
CAB	1.13	23.49	81.00	21.50	2023 [9]
C-Cz	1.14	24.59	82.12	23.02	2023 [10]
Cin-CNCs	1.15	24.84	81.07	23.18	2023 [11]
**C-Im-CN plus**	**1.20**	**25.69**	**80.45**	**24.71**	**This work**

Table S8 Fitting parameters for PL decays and derived time constants of the control and perovskite/cellulose derivatives films

No.	τ_1 (μs)	A1	τ_2 (μs)	A_2	τ_{ave} (ns)
**Control**	0.5623	444.08	0.5623	542.76	498.35
**PVSK/CA**	0.5642	447.33	0.5642	546.73	502.00
**PVSK/C-Im**	0.5591	744.36	0.5591	909.77	835.33
**PVSK/C-CN**	0.1124	301.17	0.9649	946.86	923.79
**PVSK/C-Im-CN**	0.5246	1610.51	0.5246	1968.40	1807.35

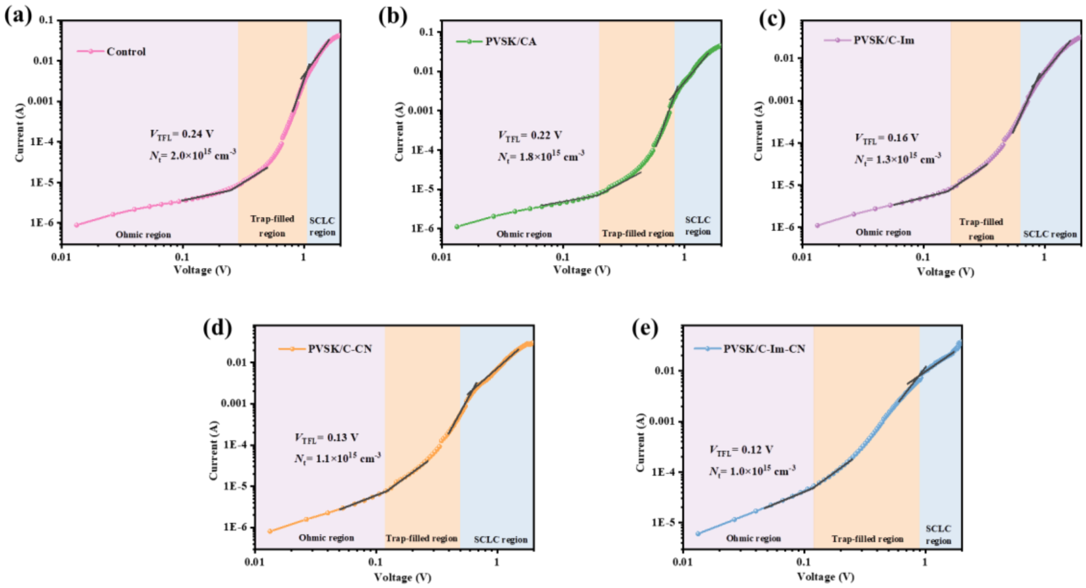

Fig. S24 Dark J-V curves for the electron-only devices with the structure of ITO/SnO_2/perovskite/PCBM/Ag

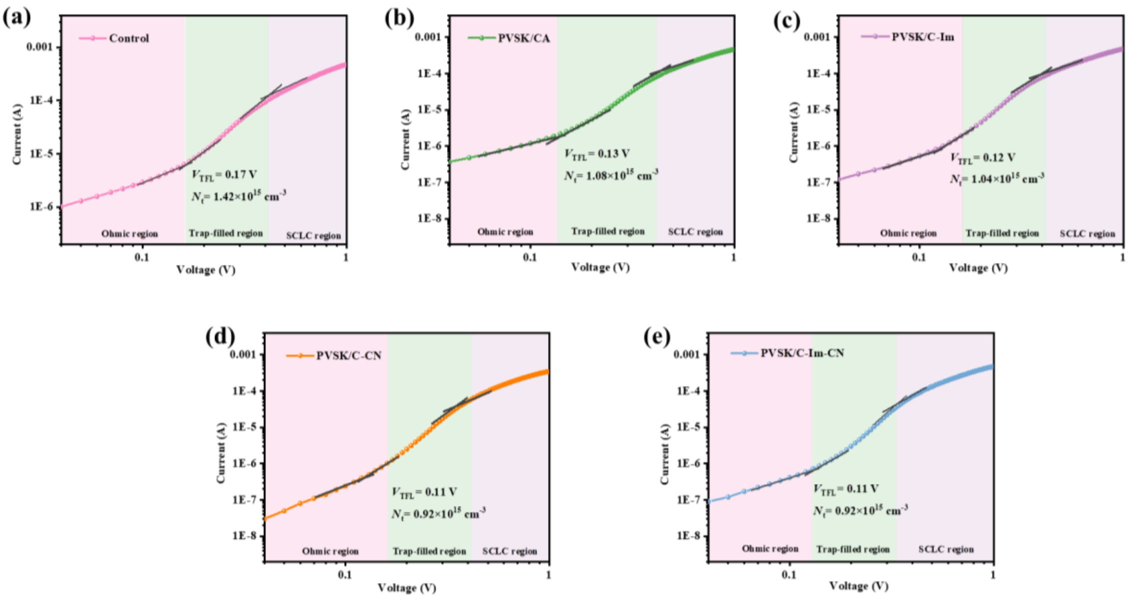

Fig. S25 Dark J-V curves for the hole-only devices with the structure of ITO/PEDOT:PSS/perovskite/Spiro-OMETAD

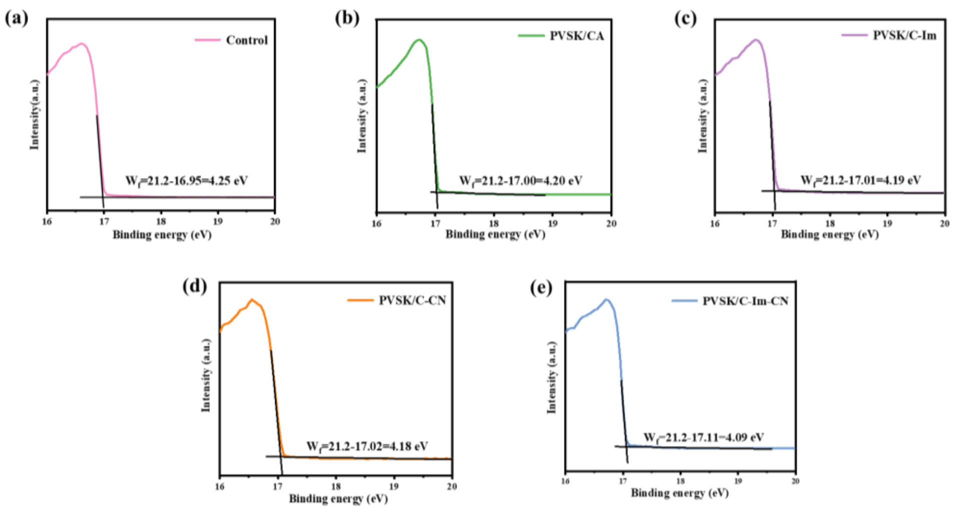

Fig. S26 UPS spectra and work function (W_f) results of the control and perovskite/cellulose derivatives films

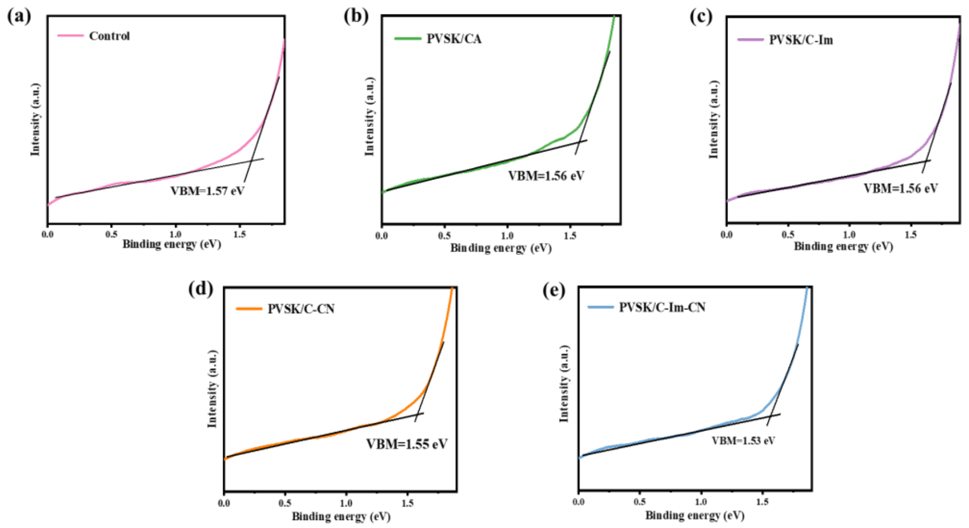

Fig. S27 UPS spectra and valence band maximum (VBM) results of the control and perovskite/cellulose derivatives films

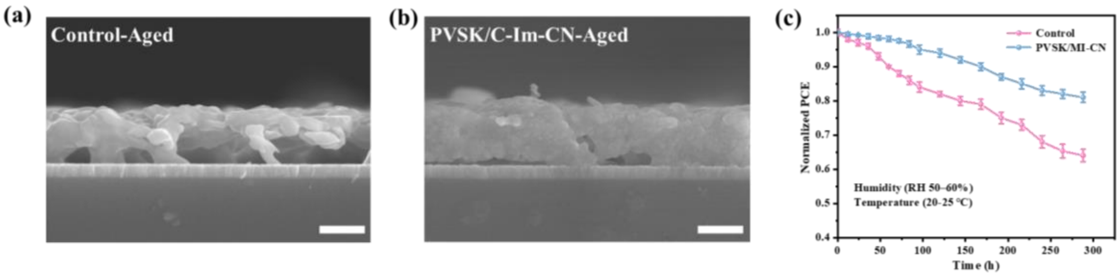

Fig. S28 (**a, b**) Cross-sectional SEM images of the control and PVSK/C-Im-CN film under high humidity condition after 300 h (RH = 50-60%, T = 20-25 °C) (Scale bar: 500 nm); (**c**) PCEs of the control and the PVSK/C-Im-CN devices under high humidity condition (RH = 50-60%, T = 20-25 °C)

Supplementary References

[S1] P. Liu, Z. Yu, N. Cheng, C. Wang, Y. Gong et al., Low-cost and efficient hole-transport-material-free perovskite solar cells employing controllable electron-transport layer based on P25 nanoparticles. Electrochimica. Acta **213**, 83–88 (2016). https://doi.org/10.1016/j.electacta.2016.07.095

[S2] J. He, C.-F. Ng, K. Young Wong, W. Liu, & T. Chen, Photostability and moisture stability of $CH_3NH_3PbI_3$ -based solar cells by ethyl cellulose. ChemPlusChem **81**, 1292–1298 (2016). https://doi.org/10.1002/cplu.201600415

[S3] J. Yang, S. Xiong, T. Qu, Y. Zhang, X. He et al., Extremely low-cost and green cellulose passivating perovskites for stable and high-performance solar cells. ACS Appl. Mater. Interfaces **11**, 13491–13498 (2019). https://doi.org/10.1021/acsami.9b01740

[S4] H.-Y. Chu, J.-Y. Hong, C.-F. Huang, J.-Y. Wu, T.-L. Wang et al., Enhanced photovoltaic properties of perovskite solar cells by the addition of cellulose derivatives to $MAPbI_3$ based photoactive layer. Cellulose **26**, 9229–9239 (2019). https://doi.org/10.1007/s10570-019-02724-2

[S5] F. Bisconti, A. Giuri, L. Dominici, S. Carallo, E. Quadrivi et al., Managing transparency through polymer/perovskite blending: A route toward thermostable and highly efficient, semi-transparent solar cells. Nano Energy **89**, 106406 (2021). https://doi.org/10.1016/j.nanoen.2021.106406

[S6] J. Liu, Q. He, J. Bi, M. Lei, W. Zhang et al., Remarkable quality improvement of $CsPbIBr_2$ perovskite film by cellulose acetate addition for efficient and stable carbon-based inorganic perovskite solar cells. Chem. Eng. J. **424**, 130324 (2021). https://doi.org/10.1016/j.cej.2021.130324

[S7] P. Zhang, N. Gu, L. Song, W.-H. Chen, P. Du et al., Bifunctional green cellulose derivatives employed for high efficiency and stable perovskite solar cells under ambient environment. J. of Alloys Compound. **886**, 161247 (2021). https://doi.org/10.1016/j.jallcom.2021.161247

[S8] X. Zhu, R. Lin, H. Gu, H. Hu, Z. Liu et al., Ecofriendly hydroxyalkyl cellulose additives for efficient and stable $MAPBI_3$ -based inverted perovskite solar cells. Energy Environ. Mater. e12426 (2022). https://doi.org/10.1002/eem2.12426

[S9] B. Xiao, Y. Qian, X. Li, Y. Tao, Z. Yi et al., Enhancing the stability of planar perovskite solar cells by green and inexpensive cellulose acetate butyrate. J. Energy Chem. **76**, 259–265 (2023). https://doi.org/10.1016/j.jechem.2022.09.039

[S10] Z. Zhang, C. Wang, F. Li, L. Liang, L. Huang et al., Bifunctional cellulose interlayer enabled efficient perovskite solar cells with simultaneously enhanced efficiency and stability. Adv. Sci. 2207202 (2023). https://doi.org/10.1002/advs.202207202

[S11] J. Liu, N. Liu, G. Li, Y. Wang, Z. Wang et al., Cinnamate-functionalized cellulose nanocrystals as interfacial layers for efficient and stable perovskite solar cells. ACS Appl. Mater. Interfaces **15**, 1348–1357 (2023). https://doi.org/10.1021/acsami.2c19193
